# The population structure of *Clostridium tetani* deduced from its pan-genome

**DOI:** 10.1038/s41598-019-47551-4

**Published:** 2019-08-02

**Authors:** Diana Chapeton-Montes, Lucile Plourde, Christiane Bouchier, Laurence Ma, Laure Diancourt, Alexis Criscuolo, Michel Robert Popoff, Holger Brüggemann

**Affiliations:** 1 Bacterial Toxins, Institut Pasteur, Paris, France; 2 Sanofi-Pasteur, Marcy l′Etoile, France; 3 Genomic Platform, Biomics, Institut Pasteur, Paris, France; 4 CNR Bactéries anaérobies Botulisme, Institut Pasteur, Paris, France; 5 Hub Bioinformatique Biostatistique, Institut Pasteur, Paris, France; 6 Aarhus University, Department of Biomedicine, Aarhus, Denmark

**Keywords:** Genetics, Bacteriology

## Abstract

*Clostridium tetani* produces a potent neurotoxin, the tetanus neurotoxin (TeNT) that is responsible for the worldwide neurological disease tetanus, but which can be efficiently prevented by vaccination with tetanus toxoid. Until now only one type of TeNT has been characterized and very little information exists about the heterogeneity among *C*. *tetani* strains. We report here the genome sequences of 26 *C*. *tetani* strains, isolated between 1949 and 2017 and obtained from different locations. Genome analyses revealed that the *C*. *tetani* population is distributed in two phylogenetic clades, a major and a minor one, with no evidence for clade separation based on geographical origin or time of isolation. The chromosome of *C*. *tetani* is highly conserved; in contrast, the TeNT-encoding plasmid shows substantial heterogeneity. TeNT itself is highly conserved among all strains; the most relevant difference is an insertion of four amino acids in the C-terminal receptor-binding domain in four strains that might impact on receptor-binding properties. Other putative virulence factors, including tetanolysin and collagenase, are encoded in all genomes. This study highlights the population structure of *C*. *tetani* and suggests that tetanus-causing strains did not undergo extensive evolutionary diversification, as judged from the high conservation of its main virulence factors.

## Introduction

Tetanus is a worldwide neurological disease of man and animals, characterized by spastic paralysis of skeletal muscles. Neonatal tetanus is currently the most prevalent form in humans with estimated 34,000 deaths in 2015^1^. The disease is caused by the tetanus toxin, solely produced by the Gram-positive, anaerobic, spore-forming species *C*. *tetani*, spores of which are widespread in the environment where they survive for long periods of time.

The tetanus toxin (TeNT) gene *tent* has been cloned and sequenced in 1986^2,3^, and it has been found to be localized on a large plasmid^4^. The mode of action was characterized, i.e. the TeNT metalloprotease activity towards the SNARE protein VAMP/synaptobrevin^5,6^ and its axonal retrograde transport to the central nervous system was analyzed in detail^7,8^. More recently, nidogens were found to mediate TeNT binding to neuronal cells and subsequent internalization into neurons^7^.

Tetanus is a preventable disease and immunization with tetanus toxoid-containing vaccines are safe and confer efficient and long-term protection^9^. Large scale worldwide vaccination programs are supported by the World Health Organization to prevent tetanus^9^. Only one TeNT type is known which is currently used for vaccine preparation. The high efficiency of this vaccine suggests that all tetanus-causing *C*. *tetani* strains produce an identical or very similar toxin. Investigations on possible TeNT variations are based on genetic and genomic analyses of *C*. *tetani* strains. The first whole genome sequence of the toxigenic strain E88 was reported in 2003^10^. Up to now, only 12 strains have been sequenced at present^11–13^. The study of Cohen *et al*. focuses on U.S. vaccine strains of *C*. *tetani*, but no larger effort was undertaken to assess genomic diversity among tetanus-causing strains from various geographic locations and isolated at different times. Here we report the genomic analysis of 26 newly sequenced *C*. *tetani* strains from recent and ancient periods. Our work highlights the conservation of the chromosome and the high heterogeneity of the toxin-encoding plasmid, as well as reveals the population structure of the species with two phylogenetically distinct clades. In addition, we found that albeit a strong conservation of TeNT sequences, five out of 38 *C*. *tetani* strains encode a toxin containing four additional amino acids in the receptor-binding domain that might impact the receptor-binding properties and antigenicity.

## Results

### Strain selection and whole genome sequencing

Whole genome sequencing (WGS) was performed for 26 *C*. *tetani* strains. These including 11 strains from the National Reference Center (NRC) of Anaerobic Bacteria (Institut Pasteur, France), isolated in the time period 1984–2017, two strains from Sanofi (France), and 13 strains from the Prevot’s collection (Institut Pasteur, France), isolated in the time period 1955–1965; an exception was the Harvard strain, which was isolated before 1949 (Table 1). Most of the strains have been isolated from human wounds, except one strain from cheese and one from a cat wound. WGS of these strains resulted in 26 draft genomes with 30 to 68 contigs (on average 46 contigs). The genome size varied from 2.735 to 2.951 Mb, on average 2.844 Mb, which is almost identical to the average size of the 12 previously sequenced *C*. *tetani* genomes (2.841 Mb; Table 2).

**Table 1:** Newly sequenced *Clostridium tetani* strains of this study and previously sequenced strains, their originand sequencing data.

Strain	Clade	Genome (Mbp)	Sequencing coverage	Contigs	N50 (in kb)	CDSs*	Plasmid (kp)	TeNT	Origin, strain collection**	Year	Geographical origin
Harvard	1A	2.839	176	39	263	2,810	73.5 (single contig)	yes	PC	1949	North America
Strain 3	1A	2.839	254	39	264	2,807	ca. 73.3	yes	PC	1955	Denmark
4784 A	1A	2.836	323	45	155	2,809	ca. 72.7	yes	PC	1968	
1586-U1	1A	2.808	381	42	199	2,734	ca. 72.7	yes	Sanofi	1969	France
1586-Z1	1A	2.735	632	42	199	2,654	no	no	Sanofi	1969	France
641-84	1A	2.813	277	34	271	2,749	ca. 72.4	yes	human, NRC	1984	France
46-1-08	1A	2.889	194	50	158	2,834	ca. 72.4	yes	human, NRC	2008	France
407-86	1A	2.810	326	44	222	2,786	no	no	human, NRC	1986	France
75-97	1A	2.896	236	45	222	2,864	ca. 72.5	yes	human, NRC	1997	France
89-12	1A	2.900	462	41	200	2,870	ca. 72.8	yes	NRC, human (tumefaction)	2012	France
TMB2	1B	2.807	473	30	319	2,777	69.3 (single contig)	yes	human, PC	1956	France
B4	1 C	2.818	279	40	248	2,757	ca. 61.7	yes	PC	1962	
1240	1 C	2.804	211	51	201	2,744	ca. 59.9	yes	cat wound, PC	1955	France
1337	1 C	2.817	236	44	271	2,755	ca. 61.6	yes	PC	1955	France
3582	1D	2.945	399	42	230	2,868	ca. 91.1	yes	human wound, PC	1964	France
COR1	1E	2.860	267	48	235	2,845	ca. 78.2	no	Human (uterus perforation), PC	1955	France
329	1E	2.861	243	50	235	2,850	ca. 67.9	no	PC	1965	URSS
157-15	1E	2.762	453	40	215	2,683	ca. 52.6	no	human (M 30 y), femur fracture NRC	2014	France
512-15	1F	2.866	132	52	130	2,781	ca. 69.3	yes	cheese, PC	1955	Vietnam
358-99	1F	2.811	207	56	130	2,726	ca. 53.7	yes	human, NRC	1999	France
202-15	1F	2.816	407	68	146	2,743	ca. 67.5	yes	human (F 49 y), fracture/osteomyelitis, NRC	2015	France
132CV	1G	2.808	122	63	147	2,723	63.2 (single contig)	yes	PC	1955	Germany
2017-061	1H	2.838	238	42	271	2,770	ca. 58.8	yes	Human (M 54 y), fracture/septic knee arthritis, NRC	2016	France
3483	2	2.937	413	66	131	2,949	75.3 (single contig)	yes	human tetanus, PC	1964	France
63-05	2	2.951	243	59	121	2,960	ca. 75.8	yes	human (F 84 y), wound tetanus, NRC	2005	France
778-17	2	2.878	289	32	237	2,799	ca. 76.7	yes	human wound, NRC	2017	France

*predicted by RAST.

**PC, Prevot’s Collection; NRC, National Reference Center for Anaerobic bacteria and Botulism, France.

M, male; F, female; y, year.

**Table 2:** Previously sequenced strains.

Strain	clade	Genome (Mb)	contigs	N50 (in kb)	CDSs*	Plasmid (kp)	TeNT	Origin, strain collection	Year	Geographical origin	Reference or accession
E88	1A	2.873	2	—	2,824	74.0 (closed)	yes	Harvard derivative	1920	North America	^10^
CN655	1A	2.850	118	110	2,819	ca. 69.7	yes	Harvard derivative		North America	^13^
strain A	1A	2.824	93	114	2,781	ca. 71.7	yes	Harvard derivative		North America	^13^
strain C2	1A	2.829	39	263	2,799	ca. 72.0	yes	unknown		North America	^12^
ATCC19406_FDA	1A	2.789	52	153	2,757	No plasmid	no	unknown		North America	^12^
ATCC19406_DOE	1A	2.789	34	146	2,757	No plasmid	no	unknown		unknown	Unpublished; FUWT
ATCC9441	1B	2.800	28	1734	2,746	80.5 (single contig)	yes	unknown		North America	^12^
ATCC454	1D	2.853	67	87	2,805	ca. 62.2	no	feces		China	Unpublished; LBNB
ATCC453	1D	2.890	40	254	2,836	ca. 90.9	yes	feces		China	^12^
Mfbjulcb2	1G	2.811	1	—	2,743	?	yes	retail fish market	2004	India	Unpublished; CP027782.1
184.08	2	2.914	152	70	2,905	ca. 73.0	yes	Human (M 75 y), septic arthritis	2008	France	^13^
12124569	2	2.866	3	2559	2,886	58.4 (closed)	yes	human (M 26 y), tibia fracture, no tetanus	2012	France	^49^

*Predicted by RAST.

### Phylogenomic analysis of *C*. *tetani*

**Figure 1:**
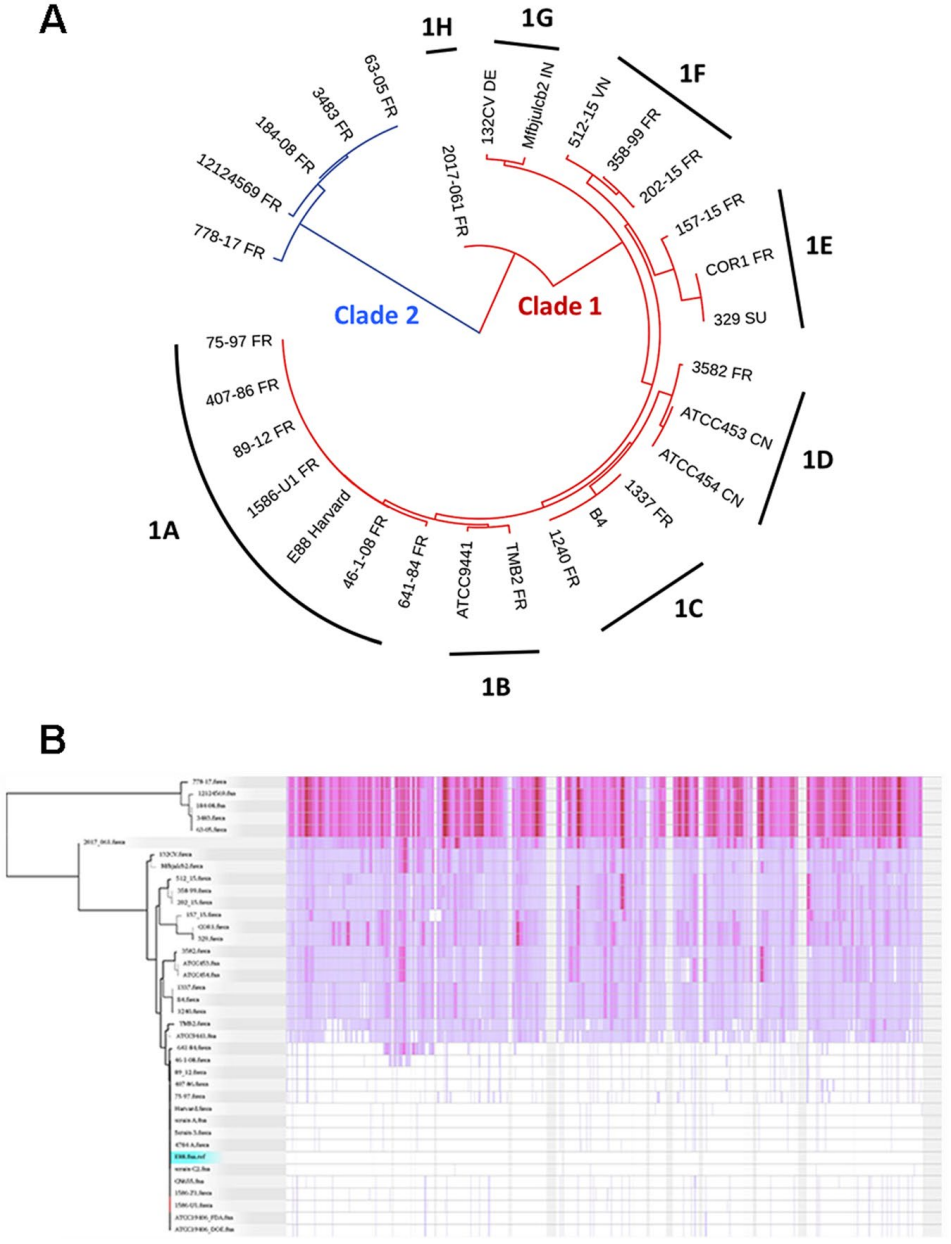
Phylogenomic comparison of *C*. *tetani* strains and visualization of SNPs in the core genome. (**A**) The phylogenetic relation was reconstructed from a core genome alignment and the comparison of high-quality SNPs of all newly and previously sequenced *C*. *tetani* strains. Two main clades exist: the major clade 1 contains most of the strains (32 strains) and the minor clade 2 contains five strains only. There is a subdivision of clade 1 strains into eight subclades (1A to 1H), with subclade 1A containing most strains. The branch length indicates the individuality of each clade, subclade and strain. The clade 1A strain E88 was taken as reference. (**B**) The core genome of *C*. *tetani* was 77% of the whole genome; high-quality SNPs were called, and visualized (as purple lines) across the genome, with strain E88 taken as reference. This comparison built the fundament for the phylogenetic reconstruction as seen in A.

The genome sequences of the 26 strains and of the previously sequenced strains (Tables 1 and 2) were phylogetically analyzed by calling single nucleotide polymorphisms (SNPs) within the core genome using the tool ParSnp. According to this analysis, the core genome comprises 77% of the reference genome (strain E88), with a total number of 94,816 SNPs (Fig. 1). The SNP analysis revealed that *C*. *tetani* strains can be separated into two clades: clade 1 comprises the large majority of strains (33 strains), and clade 2, as a minor clade, comprises five strains (strains 778-17, 12124569, 184-08, 3483 and 63-05) (Fig. 1). Clade 1 strains can be further distinguished based on their SNPs into eight subclades (1A to 1H). About half of all clade 1 strains belong to the subclade 1A (15 strains), including the Harvard/Massachusetts-derived strains that are used in vaccine production and more recent isolates. These subclade 1A strains include strain A, E88, CN655, and ATCC19406. They all originate from strain Harvard, and are thus redundant. Among subclade 1A strains are also more recently isolated strains, such as strain C2, 4784-A, strain 3, 1586, that are independent isolates from the Prevot’s collection, and strains 75–97 and 407-86 that are more recent isolates from the anaerobic laboratory at the Institut Pasteur. Subclade 1A strains are highly similar, with only 292 SNPs in total (Fig. 1). The other subclades within clade 1 comprise one to three strains only. Subclades within clade 1 that are most distant to subclade 1A are subclade 1G, containing two strains, including an Indian isolate (strain Mfbjulcb2), and subclade 1 H, containing only strain 2017.061; this strain could represent a hybrid of clade 1 and 2 strains, or a founder of these two clades.

The phylogenomic analysis did not reveal an obvious separation of strains based on their geographical origin, disease association or isolation time, since recently isolated strains and those isolated 50–70 years ago are distributed in different (sub)clades.

### Diversity of the tetanus toxin-encoding plasmid

**Figure 2:**
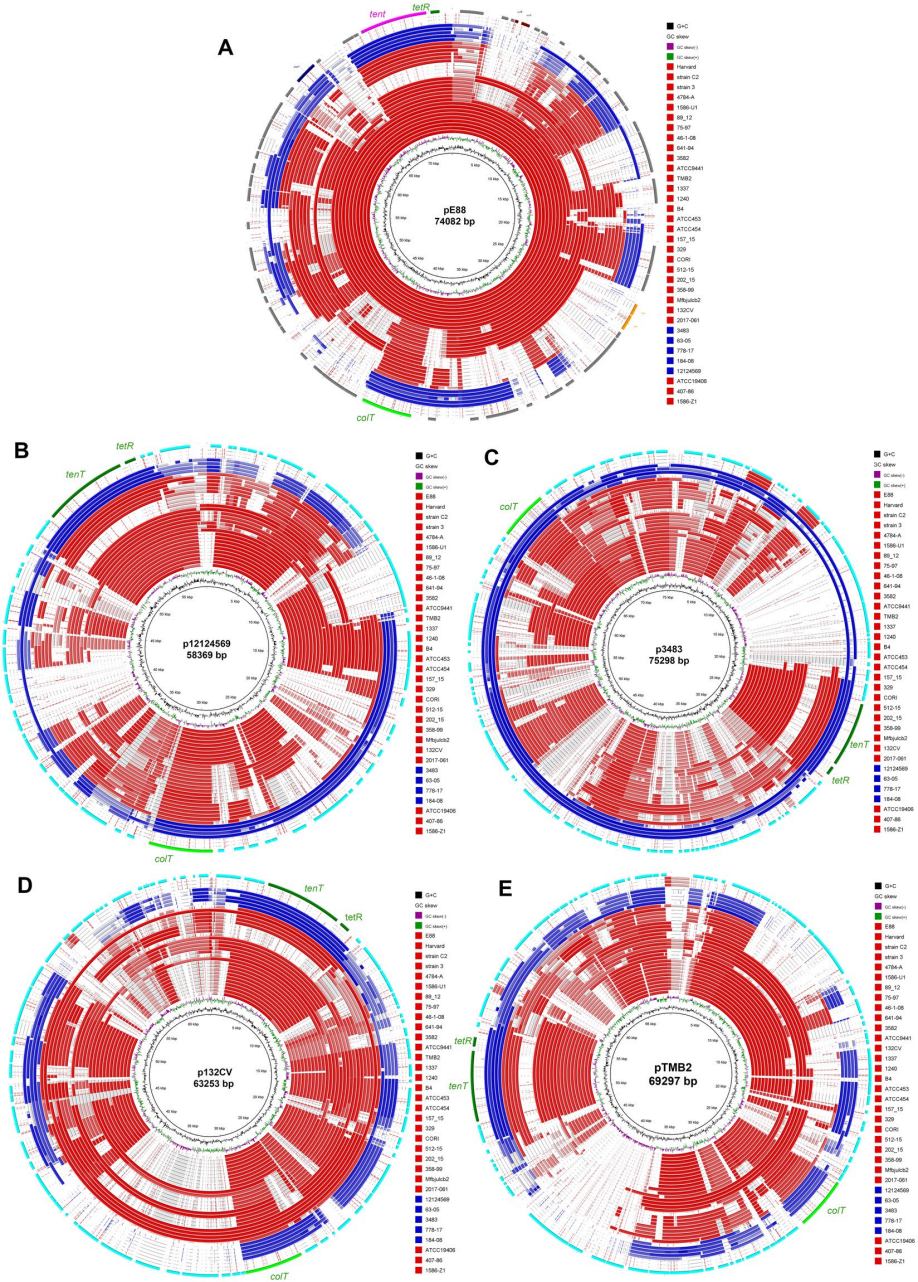
Comparison of the large tetanus toxin-encoding plasmids. (**A**) The closed plasmid pE88 of clade 1A strain E88 was used as reference. All other *C*. *tetani* genomes were searched against p88. It can be seen that all clade 1A genomes shared the same or a highly similar plasmid, except three strains (ATCC19406, 1586-Z1 and 407-86). The most outer ring depicts genes identified on pE88; important genes are highlighted in color, including *tent* (in pink) and *colT* (in light green). All other strains (subclades 1B to 1H and clade 2) carried substantial differences in their plasmids. The following references were taken: (**B**) the plasmid in clade 2 strain 12124569; (**C**) the plasmid contig in clade 2 strain 3483; (**D**) the plasmid contig in subclade 1G strain 132CV; (**E**) the plasmid contig in subclade 1B strain TMB2. Only comparisons are shown for strains, for which the plasmid sequence was present in a single contig, likely corresponding to the entire plasmid sequence.

All draft genomes were searched against the circular tetanus toxin-encoding plasmid pE88 from strain E88 (Brüggemann *et al*., 2003). This revealed that the plasmid sequence was not conserved in most strains. Only subclade 1A strains contained a plasmid identical to, or highly similar with pE88 (Fig. 2A). Exceptions were three subclade 1A strains (ATCC19406, 1586-Z1 and 407-86): no plasmid sequences were detected in the genome assemblies of these strains.

All other subclades of clade 1 as well as clade 2 strains contained plasmids that differed from pE88 (Fig. 2B–E). Even within some subclades of clade 1, i.e. subclades 1B, 1 C and 1D, strains differed regarding their plasmid sequences. Most distant to pE88 were the plasmids of clade 2 strains, as well as strains of subclades 1E to 1 H. The estimated plasmid sizes in these strains varied from 52.6 kb to 78.2 kb. This is an estimation, based on the sum of all contigs that showed similarity to previously sequenced *C*. *tetani* plasmids. For four strains (one strain of each subclade 1A, 1B, 1G and clade 2), a single contig was found that corresponds very likely to the complete plasmid (Table 1).

**Figure 3:**
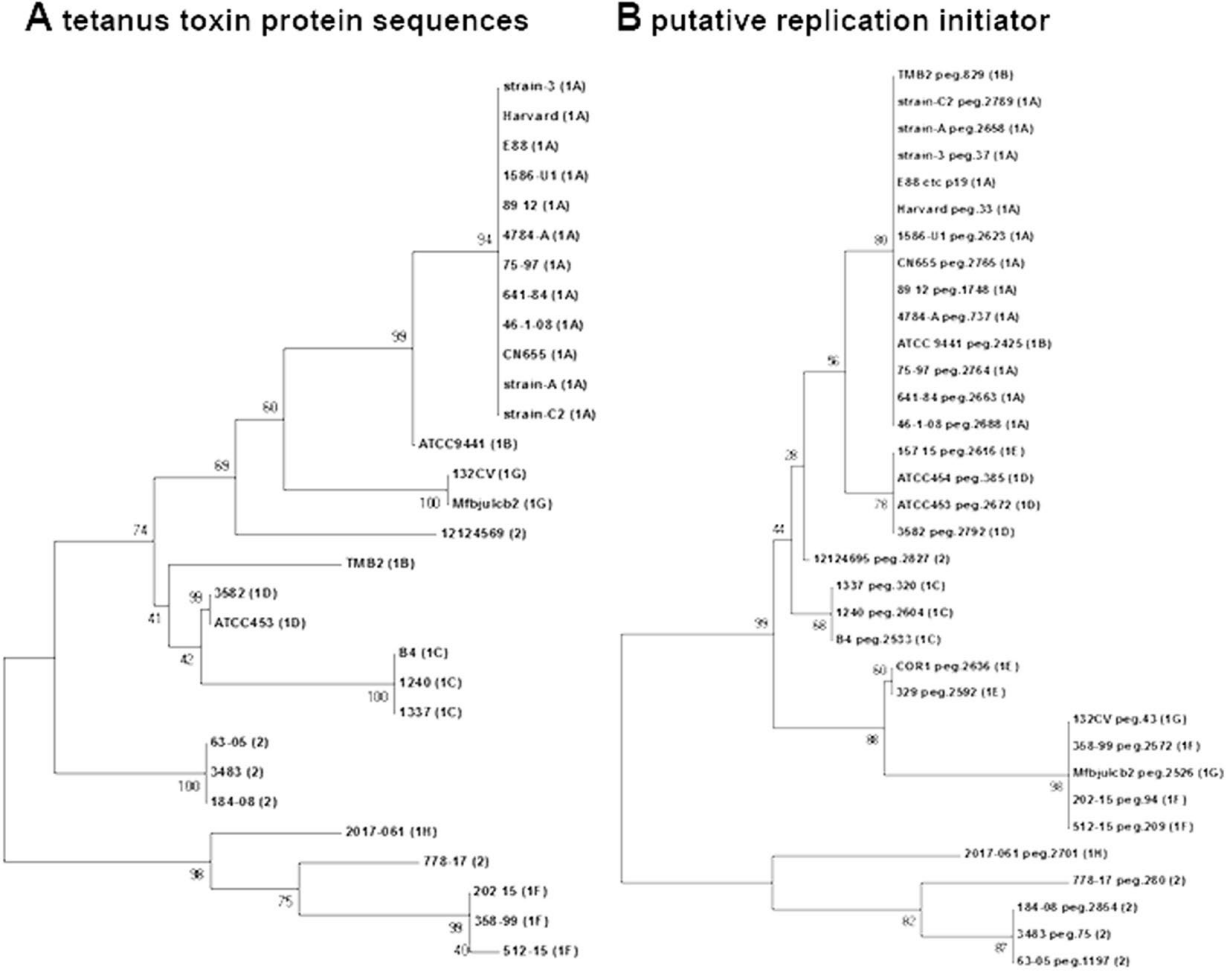
Phylogenetic comparison of plasmid-encoded conserved proteins. The protein sequences of the (**A**) tetanus toxin and (**B**) putative replication initiator were extracted from all genomes and phylogenetically compared. For all strains, the clade and subclade assignments are given in brackets. Regarding TeNT variants, five strains (all three subclade 1F strains, the subclade 1H strain 2017.061 and one type 2 strain (778-17) carry most diverged variants, that harbor a four-amino-acid insertion in the C-terminal domain (see Figs S1 and S2).

Next, we searched the plasmid sequences for the most important plasmid genes, i.e. the toxin gene *tent* and the upstream regulatory gene *tetR* which encodes for an alternative sigma factor^14^. The *tent* and *tetR* genes were found in all plasmid-carrying strains, except for the three strains of subclade 1E and one strain of subclade 1D (ATCC454). The plasmid in the latter strain was exceptional, showing a large deletion. TeNT was identical on protein level in all subclade 1A strains; in all other strains there were a few variations that corresponded largely with the different subclades (Fig. 3A). Strains of subclade 1 F carried the most distant TeNT variant compared to subclade 1A. Strains of subclade 1 F, 1 H, as well as clade 2 strain 778.17 encode a TeNT with four amino acids in the C-terminal domain (Fig. S1). This amino acid insertion has been confirmed by PCR amplification of the 3′ part of *tent* and subsequent sequencing (Fig. S2). Regarding additional *tent*-regulating elements, the plasmid of clade 1A strains encodes a two-component system (TCS), which has been found to positively control TeNT synthesis (manuscript in preparation); the TCS genes are present only in clade 1 strains of the subclades 1A, 1B and 1 C.

Interestingly, all plasmid-carrying strains, including the clade 2 strains contained the gene *colT*, encoding a collagenase, a putative virulence factor that is possibly involved in tissue colonization^10^. Only one additional gene was found to be conserved in all plasmids: ctc_p19, encoding a 516 amino acid protein of unknown function, but with homology to proteins of the replication initiator protein A (RPA) family, suggesting that this protein is essential for plasmid replication. A phylogenetic analysis of all ctc_p19 homologs revealed that the variation of the putative RPA corresponded largely with the separation of the different (sub)clades (Fig. 3B). This result suggests that in the majority of strains the plasmid was not recently acquired, but evolved together with the chromosome. An exception was the plasmid in the clade 2 strain 12124695, whose RPA sequence was more similar to those of clade 1 strains. The RPA sequences of the other four clade 2 strains (778-17, 184-08, 3483 and 63-05) as well as the subclade 1 H strain 2017.061 showed the most diverged RPA variant compared to subclade 1A RPA homologs. There were 13 amino acid replacements, including five radical replacements that are distributed over the entire protein length.

When the previously sequenced plasmid of the clade 2 strain 12124569 was taken as reference, it could be shown that this plasmid is not conserved among clade 2 strains (Fig. 2B). The majority of clade 2 strains possess a plasmid identical or highly similar to the one found in strain 3483 (Fig. 2C); this plasmid contains a clade 2-specific region of 9 kb downstream of the *tetR-tent* locus, encoding a type III restriction-modification (RM) system. The clade 1G strain 132CV contained a plasmid that is shared with the other clade 1G strain (Mfbjulcb2), and to some extent with strains of clades 1 F and 1D (Fig. 2D). These plasmids shared for example a 10 kb region, located in direct proximity to *colT*; it encodes a cluster of proteins with unknown functions, including proteins with predicted peptidase and transport activities. The plasmid of the clade 1B strain TMB2 contained a smaller plasmid (Fig. 2E), with a strain-specific region of 8 kb, encoding a type II restriction-modification system.

Taken together, the data show the high variability of the plasmid among *C*. *tetani* strains; however, a strong conservation can be seen regarding TeNT, TetR, ColT and the putative replication initiator protein.

### Conservation of chromosomal genes

The sequenced *C*. *tetani* genomes are similar in size (Tables 1 and 2). Annotation of the genomes resulted in 2,654 to 2,960 coding sequences (CDS) per strain. The pan-genome encodes 3,915 CDS, i.e. the total number of detected CDS in the *C*. *tetani* population (Table S1). In total, 1,266 CDS (32% of CDS) are shared by all strains, and 2,292 (58% of CDS) by 90% of all strains. There are no strain-specific genes; 411 CDS are present in two strains only, and mainly comprise phage-related CDS.

**Figure 4:**
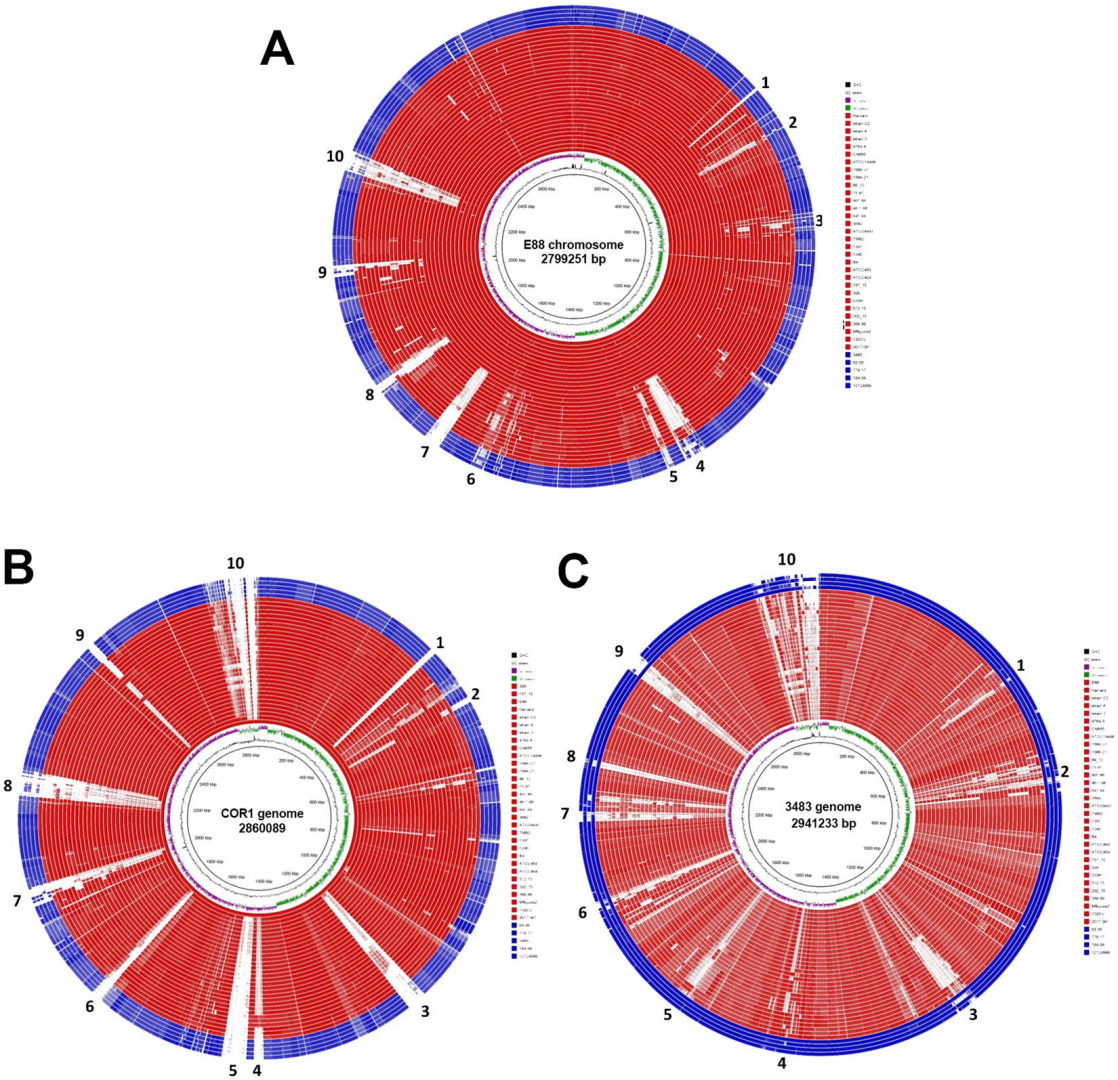
Comparison of all *C*. *tetani* genomes with strain E88, clade 1E strain COR1 and clade 2 strain 3483 as references. (**A**) The chromosome of the reference strain E88 was used. Clade 1 and 2 genomes are shown in red and blue, respectively. Ten larger (>5 kb) regions of genomic flexibility were identified; these represent/encode: 1, Phage-related mobile element; 2, putative surface-protein cluster; 3, Sigma factor locus; 4, 7 and 10, (cryptic) prophages; 5 and 6, CRISPR/cas loci; 8, iron transport system, putative phosphocholine synthesis, cell wall/spore coat/envelope/membrane modification system; 9, plasmid-like element with toxin-antitoxin system and adenine-specific methyltransferase (see also Table S2A). (**B**) The reference genome is the clade 1E strain COR1. Ten larger (>5 kb) genomic regions of flexibility were found, encoding: 1 and 9, type I restriction-modification system; 2, putative surface-protein cluster; 3, 5 and 8 (cryptic) prophages; 4, CRISPR/cas locus; 6, cell wall/spore coat/envelope/membrane modification system; 7, phage-related region with adenine-specific modification system; 10, plasmid with *colT*, but without *tent* (see also Table S2B). (**C**) The reference genome is the clade 2 strain 3483. Please note the lower nucleotide identity of clade 2 strains to clade 1 strains, represented by the fading red color. Ten larger (>5 kb) genomic regions of flexibility were found. Five regions (2,3,7,8,9) carry phage-related genes. Other regions encode: 1, putative surface-protein cluster; 4, two-component system and ABC transport system; 5, putative metabolism of aromatic compounds; 6, restriction-modification system. The plasmid (region 10) contains a type III restriction-modification system as well as lantibiotic transport system (see also Table S2C).

Clade 2 strains carry on average 118 CDS more than clade 1 strains (2,900 and 2,782 CDS, respectively; Table S1). Clade 1-specific CDS can be identified, whose genes are mostly located in genomic islands scattered around the genome (Fig. 4). Ten islands were found that are largely subclade 1A-specific, thus not only missing in clade 2 but also in subclade 1B-1H strains (Fig. 4A, Table S2A). These include three (cryptic) prophages or fragments thereof, a plasmid-like element carrying a toxin-antitoxin system, two CRISPR/cas loci, a gene cluster encoding surface-layer proteins, an iron transport system and a putative cell wall/spore coat/envelope/membrane modification system. Genomes of other clade 1 strains contained different genomic islands that carry similar functions. For example, COR1, a subclade 1E strain harbors several islands that are only shared with another subclade 1E strain (strain 329) (Fig. 4B, Table S2B); these encode functions related to restriction-modification systems, surface modification, CRISPR/cas systems and prophages. Additional smaller clusters and single genes in subclade 1E strains are predicted to encode proteins for chemotaxis, multi-antimicrobial extrusion protein (Na^+^/drug antiporter), phosphate regulon regulator and sensor (PhoR, PhoB), cell wall-binding and adhesion proteins, and DNA modification. Clade 2 genomes harbor only a few clade-specific islands, containing genes related to (pro)phages, cell surface modification, metabolism of aromatic compounds, transport and DNA methylation (Fig. 4C, Table S2C). However, there is heterogeneity among clade 2 strains, in particular regarding the presence of prophages. Apart from these genomic islands, several strain-specific genes can be found, mostly related to transposases, cell-surface/adhesion/S-layer proteins, RM systems, ABC transport systems and efflux pumps, two-component systems, resistance determinants and defense/repair functions, and specific metabolic functions (Table S1).

Since CRISPR/cas systems can contribute to the diversification within a species, we had a closer look: strain E88 and subclade 1A derivate strains contain two loci (locus numbers 5 and 6 in Fig. 4A). One locus is composed of the *cas* genes *cas6*, *csx8* (*cas8a1*), *devR* (*cas7*), *cas5*, *cas3*, *cas4*, *cas1*, *cas2* (a type I-B-like system) with a repeat consensus 5′-GTATTAGTAGCACCATATTGGAATGTAAAT-3′; the other system is composed of *cas6*, *csh1* (*cas8a*), *csh2* (*cas7*), *cas5*, *cas3*, *cas4*, *cas1* and *cas2* (another type I–B-like system) with a repeat consensus 5′-ATTTAAATACAACTCTTGTTATTGTTCAAC-3′. In a few other genomes (1E strains COR1 and 329; 1 F strains 202-15, 358-99, 512-15) there is a type II-like system (locus number 4 in Fig. 4B) with the genes *cas2*, *cas1*, *cas9* (*csn1*) harbouring the repeat consensus 5′-GTTATAGTTCCTAGTAAATTCTCCATATGCTATAAT-3′. This is in agreement with a previous study^12^.

### Additional host-interacting or virulence factors

#### Tetanolysin

All genomes carry a gene for tetanolysin (TetO), which is highly conserved; it is identical on protein level in 25 strains that all belong to clade 1. Overall, only minor variations are seen in the N-terminal part (8 amino acid changes and 11 conservative substitutions) among the 37 strains analyzed. All TetO homologs of clade 2 strains are distinct (data not shown).

#### Surface-attached proteins

**Figure 5:**
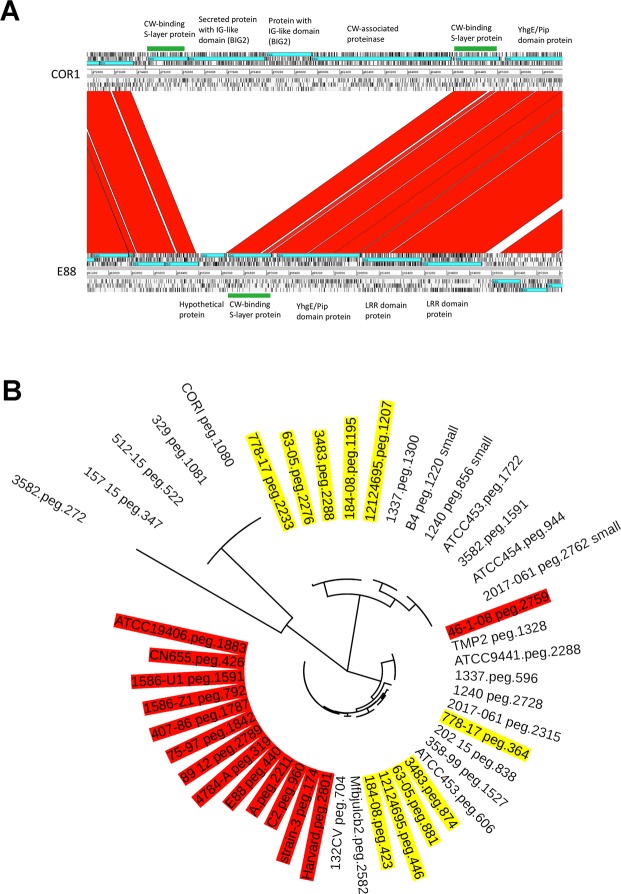
Genes encoding surface layer proteins of *C*. *tetani*: interstrain- and phylogenetic comparisons. (**A**) Comparison of gene cluster encoding surface-attached proteins in two *C*. *tetani* genomes, COR1 and E88. CDS with the characteristic clostridial cell wall-binding domain (PF04122, cell wall binding repeat 2) are highlighted in green. In total, *C*. *tetani* genomes harbor 19 or 20 proteins containing this domain. Some genomes (such as COR1 and other clade 1E genomes) carry some unique genes encoding surface-attached or secreted proteins, whose genes are inserted into the core genome. (**B**) Phylogenetic comparison of surface layer proteins: All genomes were searched against the experimentally confirmed SLP (locus tag: CTC_RS02355) of *C*. *tetani*. Three main SLP variants exist. All clade 1A (in red) and clade 2 (in yellow) genomes as well as selected strains of the other subclades carry the previously studied SLP variant, except subclade 1E strains (and also the subclade 1F strain 512-15) that carry a distantly related SLP variant. The third SLP variant is present in clade 2 strains as well as all strains from the subclades 1C, 1D and 1H.

Most clostridia contain a surface layer which is organized in a paracrystalline array surrounding the cell. Surface layers are usually composed of one or two surface-layer proteins (SLPs) or glycoproteins, which are expressed at very high levels and are anchored in the peptidoglycan of the cell wall. Clostridial SLPs have been found to retain one or several conserved motifs called clostridial cell wall binding repeat 2 (CWB2; Pfam 04122)^15^, usually located at the N-termini. In total, 19 CDSs with at least one copy of the characteristic CWB2 domain were identified in clade 1A genomes; in clade 1E and clade 2 strains 20 such CDSs are encoded per genome. The genomes of type 1E strains contain a genomic insertion that harbors genes for surface-attached proteins, including a CWB2 domain protein (Fig. 5A).

The main SLP in *C*. *tetani* was previously identified (locus tag CTC_RS02355)^16^. A BLAST search of this SLP against all other clostridial genomes showed some heterogeneity among strains, which indicates clade- and to some extent strain-specific surface structures (Fig. 5B). Clade 1A strains share a highly similar SLP; this SLP variant has three CWB2 repeats in the N-terminus and no known domain in the C-terminus. Also strains of clade 2 and clade 1B, 1G and 1 H, and most strains of clades 1 C and 1 F have this variant, with some minor substitutions in the C-terminus. However, other strains such as all clade 1E strains and some from other clades (1 C, 1D, 1 F) have different SLP variants. The difference is largely restricted to the C-terminus; interestingly, the SLP variant of clade 1E strains contains a bacterial IG-like domain in the C-terminus. In addition, every clade 2 strain has a second SLP variant; currently it is not known if both SLP variants are produced.

## Discussion

Despite a low amino acid sequence identity (21 to 41%) with botulinum neurotoxins (BoNTs), TeNT retains a common structure and enzymatic mode of action with BoNTs^17–19^. Interestingly, TeNT shows the highest level of identity (41%) with BoNT type B and both neurotoxins cleave the same substrate (synaptobrevin) at the same cleavage site^19^. It is likely that TeNT and BoNTs derive from a common ancestor gene. However, *tent* and *bont* genes have diverged in the course of evolution^20^. Indeed, *bont* is located in an operon with *ntnh* that results from a *bont* duplication and that encodes the non-toxic non-hemagglutinin (NTNH) protein^21–23^. NTNH retains a similar structure to that of BoNT but the zinc-proteolytic site is lacking^24,23^. In addition, the *ntnh-bont* operon is associated with either a hemagglutinin (*ha*) or *orfX* operon, and BoNT combines with non-toxic proteins to form complexes of various sizes (review in^25,26^). In contrast, *tent* is unique and does not show any duplication or operon association with genes encoding non-toxic proteins. Only, the regulatory gene *tetR*, that is related to *botR*, lies upstream of *tent*^14^. Moreover, in contrast to BoNTs that display large sequence variations and are distributed in 10 types and more than 40 subtypes^27,28^, TeNT shows a remarkable genetic stability in toxigenic *C*. *tetani* strains.

At the protein level, TeNT amino acid changes can be found at 50 positions, but half correspond to conservative substitutions (Fig. S1). The most striking difference is an insertion of four amino acids (‘NSES/Y’), at position 1137 of TeNT in strains of subclades 1 F, 1 H and in one clade 2 strain (778-17). This insertion lies within the C-terminal receptor binding domain (pfam07951), indicating that those strains might have different receptor-binding properties and antigenicity. The zinc-dependent proteolytic site (HELIH) as well as the residues involved in the binding to ganglioside receptor (D1222, H1271, SNWY1290, G1300, and R1226)^29^ are conserved in all TeNT sequences. An exception is H1271 that is replaced by R1271 in clade 2 strain 12124569. This indicates that all TeNT variants retain the same functional sites.

Until now, *tent* has been found only in *C*. *tetani* strains, whereas BoNTs are produced by diverse clostridial species and a few other bacterial species^27,28,30–32^. *C*. *tetani* strains show a high level of genomic conservation with a core genome of about 77%. Most of the strains (32 of 37) are distributed in one clade (clade 1) with further subdivision in eight subclades (1A to 1 H). Most strains sequenced to date are subclade 1A strains. Thus, previous knowledge about *C*. *tetani* is very much restricted to those strains; all strains used for tetanus toxoid production are clade 1A strains. Five strains, historical and recent isolates, are more distantly related and constitute a second clade (clade 2). The main genomic variations include genes of prophages, cell surface proteins, and DNA modification and defence (RM, CRISPR/cas systems). The strains of the different clades and subclades do often not share parameters such as isolation time or geographic origin. This indicates no obvious evolutionary trend over time and space.

*C*. *tetani* and BoNT-producing clostridia are spore-forming bacteria, the main habitat of which is the environment. It is intriguing to better understand the environmental factors and selective pressures involved in the high genetic diversity of BoNT-producing clostridia, in contrast to the the conservation of *C*. *tetani*, respectively.

The gene *tent* is localized on large plasmids in *C*. *tetani*, that in contrast to the chromosomes show a high variability. Interestingly, *bontB*, which encodes the most related BoNT to TeNT, is also frequently localized on plasmids in *C*. *botulinum*^33,34^. Interestingly, it was recently shown that BoNT-encoding plasmids can be transferred by conjugation between various *Clostridium* species^35^. Here, no relatedness between *C*. *tetani* and *C*. *botulinum* plasmids was observed. Phylogenetic comparison of the putative replication initiator protein homologs encoded in all *C*. *tetani* plasmids indicates coevolution of plasmid and chromosome, since a similar separation into clades and subclades can be seen for most strains (Figs 1 and S1B). This also indicates that there was no recent plasmid acquisition by horizontal gene transfer, at least not between phylogenetically distant *C*. *tetani* strains. However, several strain-specific genes and regions can be found on the plasmids of individual strains. This highlights the evolutionary more dynamic nature of the plasmid, which can serve as a sink for horizontally transferred genes.

Only few additional virulence factors are known for *C*. *tetani*, such as the chromosomally encoded tetanolysin TetO and, putatively, the plasmid-encoded collagenase ColT; these two factors are highly conserved in all strains. TetO is a pore-forming toxin, which attacks macrophages and thus facilitates the early step of wound colonization by *C*. *tetani*^36^. The N-terminal domain of cholesterol-dependent cytolysins including TetO is not directly involved in the interaction with the membrane receptor and the mechanism of pore formation; it likely stabilizes the toxin monomers and contributes to oligomer formation^37^. Thus, the eight here detected amino acid changes in this domain (data not shown) seem not to be critical for toxin activity. The collagenase ColT also shows a high level of similarity at the amino acid level (94 to 100%) (data not shown). The minor variations in ColT reflect the clade distribution of the genome.

A surface-exposed proteineous layer, consisting of regularly assembled SLPs is common in Gram-negative and Gram-positive bacteria. Most clostridial species contain one or two SLPs. For example, *C*. *difficile* produces two main SLPs, but contains many (29) cell wall proteins (CWPs), harboring the characteristic CWB2 motif^38^. *C*. *tetani* strains possess 19 or 20 CWB2-containing proteins, but some variation exists between strains that affect mostly the C-termini of such proteins, that are exposed into the extracellular space. Surface-attached proteins have multiple roles; notably, in *C*. *difficile* they have been found to mediate bacterial adhesion to the intestinal mucus layer and gastrointestinal cells as well as to induce a pro-inflammatory innate immune signaling via toll-like receptor 4 (TLR4)^39,40^. *C*. *tetani* likely uses the surface-attached proteins in wound colonization. The numerous proteins and their diversity possibly reflect specific abilities and strain-specific variations to colonize tissues and, possibly, a strategy to divert host recognition. As for other bacteria such as *C*. *difficile*^39^, SLPs could be used as vaccine candidates to prevent *C*. *tetani* colonization in addition to the tetanus toxoid.

## Materials and Methods

### Bacterial strains

The *C*. *tetani* strains used for this study are listed in Table 1. *C*. *tetani* strains were grown in TGY broth (pH 7.5) containing trypticase (Trypticase-Glucose Yeast Peptone BBL, BD Biosciences; 30 g/L), yeast extract (Bacto Yeast Extract, BD Biosciences; 20 g/L), glucose (5 g/L) and cystein, HCl (0.5 g/L) under anaerobic conditions.

### DNA extraction and genome sequencing

Genomic DNA from all strains of *C*. *tetani* was extracted and purified as previously described^41,42^. Whole genome sequencing (WGS) using the NEXTflex^®^ PCR-Free DNA-Seq kit for Illumina Plaforms (Bioo Scientific Corporation) were performed using MiSeq device (Illumina) in paired-end reads of 250 or 300 bases. Sequence files were generated using Illumina Analysis Pipeline version 1.8 (CASAVA). Reads were trimmed using a quality control pipeline^43^. The assembly of sequence reads was performed using SOAPdenovo (version 1.05).

### Genome comparison, phylogenomic and other bioinformatic analyses

For phylogenomic analyses, the core genome was identified and aligned with Parsnp, a program that is part of the Harvest software package^44^. Parsnp aligns microbial genomes based on a suffix graph data structure; the output is a core-genome alignment that contains all SNPs, Indels, and structural variation within the core genome. Parsnp is further quality-filtering SNPs; only reliable core-genome SNPs are considered for reconstruction of the whole-genome phylogeny that can be visualized with Gingr, another program of the Harvest software package^44^.

Gene prediction and annotation of all genomes were performed with RAST^45^. Phylogenetic trees were visualized using Mega v7^46^ and Interactive Tree Of Life (iTOL; https://itol.embl.de/). For comparative genome analyses and visualization, the program BRIG was used^47^. To determine orthologous genes among the *C*. *tetani* strains we used the tool Proteinortho^48^.

### Sequence accession

The genome sequence accession numbers are: 1240, QMBG00000000; 132CV, QMAZ00000000; 1337, QMAN00000000; 157-15, QMAR00000000; 1586-U1, QMBI00000000; 1586-Z1, QMBH00000000; 2017-061, QMAP00000000; 202-15; QMAQ00000000; 329, QMBF00000000; 3483, QMDR00000000; 3582, QMBA00000000; 358-99, QMAV00000000; 407-86, QMAX00000000; 46-1-08, QMAT00000000; 4784A, QMBJ00000000; 512-15, QMBE00000000; 63-05, QMAU00000000; 641-84, QMAY00000000; 75-97, QMAW00000000; 778-17, QMAO00000000; 89-12, QMAS00000000; B4, QMBD00000000; COR1, QMBC00000000; Harvard, QMBL00000000; Strain-3, QMBK00000000; TMB2, QMBB00000000.

Previously sequenced strains have these accession numbers: E88, AE015927.1 (chromosome) and AF528097.1 (plasmid); CN655, JSWC00000000; strain A, JWIX00000000; strain C2, JRGG00000000; ATCC19406, JRGJ00000000 and FUWT00000000; ATCC9441, JRGH00000000; ATCC454, LBNB00000000; ATCC453, JRGI00000000; Mfbjulcb2, CP027782.1; 184.08, JSWD00000000; 12124569, HG530135.1 (chromosome) and HG530136.1 (plasmid).

## Supplementary information

Table S1

Table S2 Fig S1 S2

## References

[1] Burgess C (2017). Eliminating maternal and neonatal tetanus and closing the immunity gap. Lancet.

[2] Fairweather NF, Lyness VA (1986). The complete nucleotide sequence of tetanus toxin. Nucleic. Acids Res..

[3] Eisel U (1986). Tetanus toxin: primary structure, expression in *E*. *coli*, and homology with botulinum toxins. EMBO J..

[4] Finn CW (1984). The structural gene for tetanus neurotoxin is on a plasmid. Science.

[5] Schiavo G (1992). Tetanus toxin is a zinc protein and its inhibition of neurotransmitter release and protease activity depend on zinc. EMBO J..

[6] Schiavo G, Rossetto O, Santucci A, DasGupta BR, Montecucco C (1992). Botulinum neurotoxins are zinc proteins. J. Biol. Chem..

[7] Bercsenyi K (2014). Tetanus toxin entry. Nidogens are therapeutic targets for the prevention of tetanus. Science.

[8] Surana S (2018). The travel diaries of tetanus and botulinum neurotoxins. Toxicon.

[9] WHO (2017). Tetanus vaccines: WHO position paper - February 2017. Wkly Epidemiol Rec.

[10] Brüggemann H (2003). The genome sequence of *Clostridium tetani*, the causative agent of tetanus disease. Proc. Ntl. Acad. Sci. (USA).

[11] Fournier PE (2014). Genome of a chronic osteitis-causing Clostridium tetani. New Microbes New Infect.

[12] Cohen JE, Wang R, Shen RF, Wu WW, Keller JE (2017). Comparative pathogenomics of Clostridium tetani. PLoS One.

[13] Bruggemann H (2015). Genomics of Clostridium tetani. Res Microbiol.

[14] Marvaud JC, Eisel U, Binz T, Niemann H, Popoff MR (1998). *tetR* is a positive regulator of the Tetanus toxin gene in *Clostridium tetani* and is homologous to *botR*. Infect. Immun..

[15] Fagan RP (2011). A proposed nomenclature for cell wall proteins of Clostridium difficile. J Med Microbiol.

[16] Qazi O (2007). Identification and characterization of the surface-layer protein of Clostridium tetani. FEMS Microbiol Lett.

[17] Masuyer G, Conrad J, Stenmark P (2017). The structure of the tetanus toxin reveals pH-mediated domain dynamics. EMBO Rep.

[18] Minton NP (1995). Molecular genetics of clostridial neurotoxins. Curr. Top. Microbiol. Immunol..

[19] Schiavo G (1992). Tetanus and Botulinum-B neurotoxins block neurotransmitter release by proteolytic cleavage of synaptobrevin. Nature (London).

[20] Mansfield MJ, Doxey AC (2018). Genomic insights into the evolution and ecology of botulinum neurotoxins. Pathog Dis..

[21] Doxey AC, Lynch MD, Muller KM, Meiering EM, McConkey BJ (2008). Insights into the evolutionary origins of clostridial neurotoxins from analysis of the *Clostridium botulinum* strain A neurotoxin gene cluster. BMC Evol Biol.

[22] Popoff MR, Bouvet P (2013). Genetic characteristics of toxigenic Clostridia and toxin gene evolution. Toxicon.

[23] Inui K (2012). Toxic and nontoxic components of botulinum neurotoxin complex are evolved from a common ancestral zinc protein. Biochem Biophys Res Commun.

[24] Gu S (2012). Botulinum neurotoxin is shielded by NTNHA in an interlocked complex. Science.

[25] Poulain, B., Molgo, J. & Popoff, M. R. In *The Comprehensive Sourcebook of Bacterial Protein Toxins* (eds J. Alouf, D. Ladant, & M. R. Popoff) Ch. 11, 287–336 (Elsevier, 2015).

[26] Gu S, Jin R (2013). Assembly and function of the botulinum neurotoxin progenitor complex. Curr Top Microbiol Immunol.

[27] Peck MW (2017). Historical Perspectives and Guidelines for Botulinum Neurotoxin Subtype Nomenclature. Toxins (Basel).

[28] Doxey AC, Mansfield MJ, Montecucco C (2018). Discovery of novel bacterial toxins by genomics and computational biology. Toxicon.

[29] Rummel A (2017). Two Feet on the Membrane: Uptake of Clostridial Neurotoxins. Curr Top Microbiol Immunol.

[30] Mansfield MJ, Adams JB, Doxey AC (2015). Botulinum neurotoxin homologs in non-Clostridium species. FEBS Lett.

[31] Popoff MR (2018). Botulinum Neurotoxins: Still a Privilege of Clostridia?. Cell Host Microbe.

[32] Zhang S (2018). Identification of a Botulinum Neurotoxin-like Toxin in a Commensal Strain of *Enterococcus faecium*. Cell Host Microbe.

[33] Carter AT, Austin JW, Weedmark KA, Corbett C, Peck MW (2014). Three classes of plasmid (47-63 kb) carry the type B neurotoxin gene cluster of group II *Clostridium botulinum*. Genome Biol Evol..

[34] Franciosa G, Maugliani A, Scalfaro C, Aureli P (2009). Evidence that plasmid-borne botulinum neurotoxin type B genes are widespread among *Clostridium botulinum* serotype B strains. PLoS One.

[35] Nawrocki EM, Bradshaw M, Johnson EA (2018). Botulinum neurotoxin-encoding plasmids can be conjugatively transferred to diverse clostridial strains. Sci Rep.

[36] Keyel PA, Heid ME, Salter RD (2011). Macrophage responses to bacterial toxins: a balance between activation and suppression. Immunol Res..

[37] Tweten RK, Hotze EM, Wade KR (2015). The Unique Molecular Choreography of Giant Pore Formation by the Cholesterol-Dependent Cytolysins of Gram-Positive Bacteria. Annu Rev Microbiol.

[38] Fagan RP, Fairweather NF (2014). Biogenesis and functions of bacterial S-layers. Nat Rev Microbiol.

[39] Pechine S, Bruxelle JF, Janoir C, Collignon A (2018). Targeting Clostridium difficile Surface Components to Develop Immunotherapeutic Strategies Against *Clostridium difficile* Infection. Front Microbiol.

[40] Mori N, Takahashi T (2018). Characteristics and Immunological Roles of Surface Layer Proteins in Clostridium difficile. Ann Lab Med.

[41] Popoff MR, Guillou JP, Carlier JP (1985). Taxonomic position of lecithinase-negative strains of *Clostridium sordellii*. J. Gen. Microbiol..

[42] Dineen SS, Bradshaw M, Johnson EA (2003). Neurotoxin gene clusters in *Clostridium botulinum* type A strains: sequence comparison and evolutionary implications. Cur. Microbiol..

[43] Desvillechabrol D (2018). Sequanix: a dynamic graphical interface for Snakemake workflows. Bioinformatics.

[44] Treangen TJ, Ondov BD, Koren S, Phillippy AM (2014). The Harvest suite for rapid core-genome alignment and visualization of thousands of intraspecific microbial genomes. Genome Biol.

[45] Aziz RK (2008). The RAST Server: rapid annotations using subsystems technology. BMC Genomics.

[46] Tamura K, Stecher G, Peterson D, Filipski A, Kumar S (2013). MEGA6: Molecular Evolutionary Genetics Analysis version 6.0. Mol Biol Evol.

[47] Alikhan NF, Petty NK, Ben Zakour NL, Beatson SA (2011). BLAST Ring Image Generator (BRIG): simple prokaryote genome comparisons. BMC Genomics.

[48] Lechner M (2011). Proteinortho: detection of (co-)orthologs in large-scale analysis. BMC Bioinformatics.

[49] Levy PY (2014). *Clostridium tetani* osteitis without tetanus. Emerg Infect Dis..

