# Author Correction: The population structure of *Clostridium tetani* deduced from its pan-genome

**DOI:** 10.1038/s41598-019-53688-z

**Published:** 2019-11-19

**Authors:** Diana Chapeton-Montes, Lucile Plourde, Christiane Bouchier, Laurence Ma, Laure Diancourt, Alexis Criscuolo, Michel Robert Popoff, Holger Brüggemann

**Affiliations:** 1 Bacterial Toxins, Institut Pasteur, Paris, France; 2 Sanofi-Pasteur, Marcy l′Etoile, France; 3 Genomic Platform, Biomics, Institut Pasteur, Paris, France; 4 CNR Bactéries anaérobies Botulisme, Institut Pasteur, Paris, France; 5 Hub Bioinformatique Biostatistique, Institut Pasteur, Paris, France; 6 Aarhus University, Department of Biomedicine, Aarhus, Denmark

Correction to: *Scientific Reports* 10.1038/s41598-019-47551-4, published online 02 August 2019

In the original version of this Article, Christiane Bouchier and Laurence Ma were incorrectly affiliated with ‘Biology des Infections, Institut Pasteur, Paris, France’. The correct affiliation is listed below.

Genomic Platform, Biomics, Institut Pasteur, Paris, France.

This error has now been corrected in the PDF and HTML versions of the Article.

