# Supplementary material for: The population structure of Clostridium tetani deduced from its pan-genome
Source: Sci Rep. 2019 Aug 2;9:11220. doi: 10.1038/s41598-019-47551-4 (PMC6677821; doi:10.1038/s41598-019-47551-4)
Supplement: Supplementary file 2 — Table S2 Fig S1 S2 [file 41598_2019_47551_MOESM2_ESM.pdf]

Table S2. Genomic islands > 5kb identified in the *C. tetani* genomes of E88 (clade 1A strain), COR1 (clade 1E strain) and 3483 (clade 2 strain).

Table S2A: Genomic islands > 5kb identified in the chromosome of *C. tetani* E88 (1A strain) and closely related subclade 1A strains

Number *	Position in E88 chromosome (and size)	Main encoded functions	absence in (sub)clades
1	380 kb (5 kb)	phage-related mobile element	1B,1C,1D,1E,1F,1G,1H, 2
2	460 kb (7 kb)	surface-layer cluster	1B,1C,1D,1E,1F,1H,1A strains 641-84 and 46-1-08
3	630 kb (6 kb)	sigma factor locus	1C, 1F
4	1140 kb (47 kb)	(cryptic) prophage	1B,1C,1D,1E,1F,2 strain 778-17
5	1206 kb (17 kb)	CRISPR/cas locus	1C,1E,1F,1H,2,1B strain TMB2
6	1580 kb (15 kb)	CRISPR/cas locus	1E,1F strain 512-15, 2 (except 12124569)
7	1663 kb (33 kb)	(cryptic) prophage	1B,1C,1D,1E,1F,1G,1H, 2
8	1808 kb (18 kb)	iron transport system and putative phosphocholine synthesis and a cell wall/spore coat/envelope/membrane modification system	1B,1C,1D (except strain 3582), 1E,1F,1G,1H, 2
9	2053 kb (10kb)	plasmid-like element with toxin-antitoxin system and adenine-specific methyltransferase	1B (except ATCC9441), 1C,1D,1E,1F,1G,1H, 2
10	2243 kb (35 kb)	(cryptic) prophage	1B,1C,1D,1E,1F,1G,1H, 2 and seven 1A strains

*corresponds to Figure 4A

Table S2B: **Genomic islands > 5kb identified in the genome of *C. tetani* COR1 (1E strain)**

Number *	Position ** in COR1 genome (and size)	Main encoded functions	absence in (sub)clades
1	360 kb (10 kb)	type I restriction-modification system	1A,1B,1C,1D,1F,1G,1H, 2
2	474 kb (10 kb)	surface protein cluster	1G,2, 1A (except 1A strains 641-84 and 46-1-08)
3	1085 kb (37 kb, with gap)	(cryptic) prophage with beta-lactamase	1A,1B,1C,1D,1F,1G,1H, 2,1E strain 157-15
4	1421 kb (17 kb)	CRISPR/cas locus	1A,1B,1C,1D,1G,1H, 2,1E strain 157-15
5	1454 kb (48 kb)	(cryptic) prophage with RNA sigma factor	1A,1B,1C,1D,1F,1G,1H, 2,1E strain 157-15
6	1757 kb (12 kb)	cell wall/spore coat/envelope/membrane modification system	1A,1B,1C,1D,1F,1G,1H, 2,1E strain 157-15
7	1984 kb (25 kb, with gap)	phage-related region with adenine-specific modification system	1A,1B,1C,1D,1F,1G,1H, 2
8	2189 kb (48 kb, with gap)	(cryptic) prophage	1A,1B,1C,1D,1F,1G,1H, 2,1E strain 157-15
9	2500 kb (14 kb)	type I restriction-modification system	1A,1B,1C,1F,1G,1H, 2,1E strain 157-15
10	2750 kb (78 kb)	plasmid with collagenase, but without TeNT	see plasmid maps (Fig. 2)

*corresponds to Figure 4B

** 'virtual' genome position in Figure 4B (the genome is not closed)

Table S2C: Genomic islands > 5kb identified in the genome of *C. tetani* 3483 (clade 2 strain)

**Number ***	**Position ** in 3483 genome (and size)**	**Main encoded functions**	**absence in (sub)clades**
1	420 kb (10 kb)	surface protein cluster	1B,1C,1D,1E,1F,1H and 1A strains 641-84 and 46-1-08
2	660 kb (14 kb)	(cryptic) prophage	1A,1B,1C,1D,1E,1F,1G,1H, 2 (except strains 63-05 and 184-08)
3	1180 kb (40 kb)	(cryptic) prophage	1B,1C,1D,1E,1F and clade 2 strain 778-17
4	1550 kb (10 kb)	Two-component system and ABC transport system	1A,1B,1D,1E
5	1770 kb (18 kb)	Metabolic functions (putative catabolism of aromatic compounds)	1A,1B,1C,1D,1E,1F,1H
6	2020 kb (19 kb)	restriction-modification system	1A,1B,1C,1D,1E,1F,1G,1H, 2 (except strains 63-05 and 184-08)
7	2200 kb, 2240 kb (18 kb + 13 kb)	(cryptic) prophage	1A,1B,1C,1D,1E,1F,1G,1H, 2 (except strains 63-05 and 184-08)
8	2310 kb (14 kb)	(cryptic) prophage	1A,1B,1C,1D,1E,1F,1G,1H, and clade 2 strain 778-17
9	2510 kb (34 kb)	(cryptic) prophage	1A,1B,1C,1D,1E,1F,1G,1H, 2 (except strain 63-05)
10	2840 kb (75 kb)	Plasmid with type III restriction-modification system and lantibiotic transport system	see plasmid maps (Fig. 2)

*corresponds to Figure 4C

** ‘virtual’ genome position in Figure 4C (the genome is not closed)

Figure S1: **Alignment of the tetanus toxin (TeNT) protein sequence of all so far sequenced *C. tetani* strains.** Identical TeNT sequences are represented by one strain only; in total 12 sequence variants exist.

```
778-17_(2)           MPITINNFRYSDPVNNDTIIMMEPPYCKGLDVYYKAFKITDRIWIVPERYEFGTKPEDFN      60
2017-061_(1H)        MPITINNFRYSDPVNNDTIIMMEPPYCKGLDVYYKAFKITDRIWIVPERYEFGTKPEDFN      60
202_15_(1F)          MPITINNFRYSDPVNNDTIIMMEPPYCKGLDVYYKAFKITDRIWIVPERYEFGTKPEDFN      60
512-15_(1F)          MPITINNFRYSDPVNNDTIIMMEPPYCKGLDVYYKAFKITDRIWIVPERYEFGTKPEDFN      60
63-05_(2)            MPITINNFRYSDPVNNDTIIMMEPPYCKGLDIYYKAFKITDRIWIVPERYEFGTKPEDFN      60
TMB2_(1B)            MPITINNFRYSDPVNNDTIIMMEPPYCKGLDIYYKAFKITDRIWIVPERYEFGTKPEDFN      60
3582_(1D)            MPITINNFRYSDPVNNDTIIMMEPPYCKGLDIYYKAFKITDRIWIVPERYEFGTKPEDFN      60
1337_(1C)            MPITINNFRYSDPVNNDTIIMMEPPYCKGLDIYYKAFKITDRIWIVPERYEFGTKPEDFN      60
12124569_(2)         MPITINNFRYSDPVNNDTIIMMEPPYCKGLDIYYKAFKITDRIWIVPERYEFGTKPEDFN      60
132CV_(1G)           MPITINNFRYSDPVNNDTIIMMEPPYCKGLDIYYKAFKITDRIWIVPERYEFGTKPEDFN      60
Harvard_(1A)         MPITINNFRYSDPVNNDTIIMMEPPYCKGLDIYYKAFKITDRIWIVPERYEFGTKPEDFN      60
ATCC9441_(1B)        MPITINNFRYSDPVNNDTIIMMEPPYCKGLDIYYKAFKITDRIWIVPERYEFGTKPEDFN      60
                     *******************************:****************************

778-17_(2)           PPSSLIEGASEYYDPNYLRTDSDKDRFLQAMVKLFNRIKNNVAGEALLDKIINAIPYLGN      120
2017-061_(1H)        PPSSLIEGASEYYDPNYLRTDSDKDRFLQAMVKLFNRIKNNVAGEALLDKIINAIPYLGN      120
202_15_(1F)          PPSSLIEGASEYYDPNYLRTDSDKDRFLQAMVKLFNRIKNNVAGEALLDKIINAIPYLGN      120
512-15_(1F)          PPSSLIEGASEYYDPNYLRTDSDKDRFLQAMVKLFNRIKNNVAGEALLDKIINAIPYLGN      120
63-05_(2)            PPSSLIEGASEYYDPNYLRTDSDKDRFLQIMVKLFNRIKNNVAGEALLDKIINAIPYLGN      120
TMB2_(1B)            PPSSLIEGASEYYDPNYLRTNSDKDRFLQTMVKLFNRIKNNVAGEALLDKIINAIPYLGN      120
3582_(1D)            PPSSLIEGASEYYDPNYLRTNSDKDRFLQTMVKLFNRIKNNVAGEALLDKIINAIPYLGN      120
1337_(1C)            PPSSLIEGASEYYDPNYLRTNSDKDRFLQTMVKLFNRIKNNVAGEALLDKIINAIPYLGN      120
12124569_(2)         PPSSLIEGASEYYDPNYLRTDSDKDRFLQTMVKLFNRIKNNVAGEALLDKIINAIPYLGN      120
132CV_(1G)           PPSSLIEGASEYYDPNYLRTDSDKDRFLQTMVKLFNRIKNNVAGEALLDKIINAIPYLGN      120
Harvard_(1A)         PPSSLIEGASEYYDPNYLRTDSDKDRFLQTMVKLFNRIKNNVAGEALLDKIINAIPYLGN      120
ATCC9441_(1B)        PPSSLIEGASEYYDPNYLRTDSDKDRFLQTMVKLFNRIKNNVAGEALLDKIINAIPYLGN      120
                     ********************:******** ******************************

778-17_(2)           SYSLLDKFDTNSNSVSFNLLEQDPSGATTKSATLANLIIFGPGPVLNKNEARGIVLRVDN      180
2017-061_(1H)        SYSLLDKFDTNSNSVSFNLLEQDPSGATTKSATLANLIIFGPGPVLNKNEVRGIVLRVDN      180
202_15_(1F)          SYSLLDKFDTNSNSVSFNLLEQDPSGATTKSATLANLIIFGPGPVLNKNEVRGIVLRVDN      180
512-15_(1F)          SYSLLDKFDTNSNSVSFNLLEQDPSGATTKSATLANLIIFGPGPVLNKNEVRGIVLRVDN      180
63-05_(2)            SYSLLDKFDTNSNSVSFNLSEQDPSGATTKSAMLTNLIIFGPGPVLNKNEVRGIVLRVDN      180
TMB2_(1B)            SYSLLDKFDTNSNSVSFNLSEQDPSGATTKSAMLTNLIIFGPGPVLNKNEVRGIVLRVDN      180
3582_(1D)            SYSLLDKFDTNSNSVSFNLSEQDPSGATTKSAMLTNLIIFGPGPVLNKNEVRGIVLRVDN      180
1337_(1C)            SYSLLDKFDTNSNSVSFNLSEQDPSGATTKSAMLTNLIIFGPGPVLNKNEVRGIVLRVDN      180
12124569_(2)         SYSLLDKFDTNSNSVSFNLSEQDPSGATTKSAMLTNLIIFGPGPVLNKNEVRGIVLRVDN      180
132CV_(1G)           SYSLLDKFDTNSNSVSFNLSEQDPSGATTKSAMLTNLIIFGPGPVLNKNEVRGIVLRVDN      180
Harvard_(1A)         SYSLLDKFDTNSNSVSFNLLEQDPSGATTKSAMLTNLIIFGPGPVLNKNEVRGIVLRVDN      180
ATCC9441_(1B)        SYSLLDKFDTNSNSVSFNLLEQDPSGATTKSAMLTNLIIFGPGPVLNKNEVRGIVLRVDN      180
                     ******************* ************ *:***************.*********

778-17_(2)           KNYFPCRDGFGSIMQMAFCPEYIPTFDNVIENITSLTIGKSKYFQDPALLLMHELIHVLH      240
2017-061_(1H)        KNYFPCRDGFGSIMQMAFCPEYIPTFDNVIENITSLTIGKSKYFQDPALLLMHELIHVLH      240
202_15_(1F)          KNYFPCRDGFGSIMQMAFCPEYIPTFDNVIENITSLTIGKSKYFQDPALLLMHELIHVLH      240
512-15_(1F)          KNYFPCRDGFGSIMQMAFCPEYIPTFDNVIENITSLTIGKSKYFQDPALLLMHELIHVLH      240
63-05_(2)            KNYFPCRDGFGSIMQMAFCPEYIPTFDNVIENITSLTIGKSKYFQDPALLLMHELIHVLH      240
TMB2_(1B)            KNYFPCRDGFGSIMQMAFCPEYIPTFDNVIENITSLTIGKSKYFQDPALLLMHELIHVLH      240
3582_(1D)            KNYFPCRDGFGSIMQMAFCPEYIPTFDNVIENITSLTIGKSKYFQDPALLLMHELIHVLH      240
1337_(1C)            KNYFPCRDGFGSIMQMAFCPEYIPTFDNVIENITSLTIGKSKYFQDPALLLMHELIHVLH      240
12124569_(2)         KNYFPCRDGFGSIMQMAFCPEYIPTFDNVIENITSLTIGKSKYFQDPALLLMHELIHVLH      240
132CV_(1G)           KNYFPCRDGFGSIMQMAFCPEYIPTFDNVIENITSLTIGKSKYFQDPALLLMHELIHVLH      240
Harvard_(1A)         KNYFPCRDGFGSIMQMAFCPEYVPTFDNVIENITSLTIGKSKYFQDPALLLMHELIHVLH      240
ATCC9441_(1B)        KNYFPCRDGFGSIMQMAFCPEYVPTFDNVIENITSLTIGKSKYFQDPALLLMHELIHVLH      240
                     **********************:*************************************

778-17_(2)           GLYGMQVSSHEIIPSKQEIYMQHTYPISAEELFTFGGQDANLISIDIKNDLYEKTLNDYK      300
2017-061_(1H)        GLYGMQVSSHEIIPSKQEIYMQHTYPISAEELFTFGGQDANLISIDIKTDLYEKTLNDYK      300
202_15_(1F)          GLYGMQVSSHEIIPSKQEIYMQHTYPISAEELFTFGGQDANLISIDIKTDLYEKTLNDYK      300
512-15_(1F)          GLYGMQVSSHEIIPSKQEIYMQHTYPISAEELFTFGGQDANLISIDIKTDLYEKTLNDYK      300
```

```
63-05_(2)          GLYGMQVSSHEIIPSKQEIYMQHTYPISAEELFTFGGQDANLISIDIKNDLYEKTLNDYK   300
TMB2_(1B)          GLYGMQVSSHEIIPSKQEIYMQHTYPISAEELFTFGGQDANLISIDIKNDLYEKTLNDYK   300
3582_(1D)          GLYGMQVSSHEIIPSKQEIYMQHTYPISAEELFTFGGQDANLISIDIKNDLYEKTLNDYK   300
1337_(1C)          GLYGMQVSSHEIIPSKQEIYMQHTYPISAEELFTFGGQDANLISIDIKNDLYEKTLNDYK   300
12124569_(2)       GLYGMQVSSHEIIPSKQEIYMQHTYPISAEELFTFGGQDANLISIDIKNDLYEKTLNDYK   300
132CV_(1G)         GLYGMQVSSHEIIPSKQEIYMQHTYPISAEELFTFGGQDANLISIDIKNDLYEKTLNDYK   300
Harvard_(1A)       GLYGMQVSSHEIIPSKQEIYMQHTYPISAEELFTFGGQDANLISIDIKNDLYEKTLNDYK   300
ATCC9441_(1B)      GLYGMQVSSHEIIPSKQEIYMQHTYPISAEELFTFGGQDANLISIDIKNDLYEKTLNDYK   300
                   ************************************************.***********

778-17_(2)         AIANKLSQVTSCNDPNIDIDSYKQIYQQKYQFDKDSNGQYIVNEDKFQVLYNSIMYGFTE   360
2017-061_(1H)      AIANKLSQVTSCNDPNIDIDSYKQIYQQKYQFDKDSNGQYIVNEDKFQVLYNSIMYGFTE   360
202_15_(1F)        AIANKLSQVTSCNDPNIDIDSYKQIYQQKYQFDKDSNGQYIVNEDKFQVLYNSIMYGFTE   360
512-15_(1F)        AIANKLSQVTSCNDPNIDIDSYKQIYQQKYQFDKDSNGQYIVNEDKFQVLYNSIMYGFTE   360
63-05_(2)          AIANKLSQVTSCNDPNIDIDSYKQIYQQKYQFDKDSNGQYIVNEDKFQILYNSIMYGFTE   360
TMB2_(1B)          AIANKLSQVTSCNDPNIDIDSYKQIYQQKYQFDKDSNGQYIVNEDKFQVLYNSIMYGFTE   360
3582_(1D)          AIANKLSQVTSCNDPNIDIDSYKQIYQQKYQFDKDSNGQYIVNEDKFQILYNSIMYGFTE   360
1337_(1C)          AIANKLSQVTSCNDPNIDIDSYKQIYQQKYQFDKDSNGQYIVNEDKFQVLYNSIMYGFTE   360
12124569_(2)       AIANKLSQVTSCNDPNIDIDSYKQIYQQKYQFDKDSNGQYIVNEDKFQILYNSIMYGFTE   360
132CV_(1G)         AIANKLSQVTSCNDPNIDIDSYKQIYQQKYQFDKDSNGQYIVNEDKFQILYNSIMYGFTE   360
Harvard_(1A)       AIANKLSQVTSCNDPNIDIDSYKQIYQQKYQFDKDSNGQYIVNEDKFQILYNSIMYGFTE   360
ATCC9441_(1B)      AIANKLSQVTSCNDPNIDIDSYKQIYQQKYQFDKDSNGQYIVNEDKFQILYNSIMYGFTE   360
                   ************************************************:***********

778-17_(2)         IELGKKFNIKTRLSYFSMNHDPVKIPNLLDDTIYNDLEGFNIESKDLKSEYKGQNMRVNT   420
2017-061_(1H)      IELGKKFNIKTRLSYFSMNHDPVKIPNLLDDTIYNDLEGFNIESKDLKSEYKGQNMRVNT   420
202_15_(1F)        IELGKKFNIKTRLSYFSMNHDPVKIPNLLDDTIYNDLEGFNIESKDLKSEYKGQNMRVNT   420
512-15_(1F)        IELGKKFNIKTRLSYFSMNHDPVKIPNLLDDTIYNDLEGFNIESKDLKSEYKGQNMRVNT   420
63-05_(2)          IELGKKFNIKTRLSYFSMNHDPVKIPNLLDDTIYNDTEGFNIESKDLKSEYKGQNMRVNT   420
TMB2_(1B)          IELGKKFNIKTRLSYFSMNHDPVKIPNLLDDTIYNDTEGFNIESKDLKSEYRGQNMRVNT   420
3582_(1D)          IELGKKFNIKTRLSYFSMNHDPVKIPNLLDDTIYNDTEGFNIESKDLKSEYKGQNMRVNT   420
1337_(1C)          IELGKKFNIKTRLSYFSMNHDPVKIPNLLDDTIYNDTEGFNIESKDLKSEYRGQNMRVNT   420
12124569_(2)       IELGKKFNIKTRLSYFSMNHDPVKIPNLLDDTIYNDTEGFNIESKDLKSEYKGQNMRVNT   420
132CV_(1G)         IELGKKFNIKTRLSYFSMNHDPVKIPNLLDDTIYNDTEGFNIESKDLKSEYKGQNMRVNT   420
Harvard_(1A)       IELGKKFNIKTRLSYFSMNHDPVKIPNLLDDTIYNDTEGFNIESKDLKSEYKGQNMRVNT   420
ATCC9441_(1B)      IELGKKFNIKTRLSYFSMNHDPVKIPNLLDDTIYNDTEGFNIESKDLKSEYKGQNMRVNT   420
                   ************************************ **************:********

778-17_(2)         NAFRNVDGSGLVSKLIGLCKKIIPPTNIRENLYNRTASLTDLGGELCIKIKNEDLTFIAE   480
2017-061_(1H)      NAFRNVDGSGLVSKLIGLCKKIIPPTNIRENLYNRTASLTDLGGELCIKIKNEDLTFIAE   480
202_15_(1F)        NAFRNVDGSGLVSKLIGLCKKIIPPTNIRENLYNRTASLTDLGGELCIKIKNEDLTFIAE   480
512-15_(1F)        NAFRNVDGSGLVSKLIGLCKKIIPPTNIRENLYNRTASLTDLGGELCIKIKNEDLTFIAE   480
63-05_(2)          NAFRNVDGSGLVSKLIGLCKKIIPPTNIRENLYNRTASLTDLGGELCIKIKNEDLTFIAE   480
TMB2_(1B)          NAFRNVDGSGLVSKLIGLCKKIIPPTNIRENLYNRTASLTDLGGELCIKIKNEDLTFIAE   480
3582_(1D)          NAFRNVDGSGLVSKLIGLCKKIIPPTNIRENLYNRTASLTDLGGELCIKIKNEDLTFIAE   480
1337_(1C)          NAFRNVDGSGLVSKLIGLCKKIIPPTNIRENLYNRTASLTDLGGELCIKIKNEDLTFIAE   480
12124569_(2)       NAFRNVDGSGLVSKLIGLCKKIIPPTNIRENLYNRTASLTDLGGELCIKIKNEDLTFIAE   480
132CV_(1G)         NAFRNVDGSGLVSKLIGLCKKIIPPTNIRENLYNRTASLTDLGGELCIKIKNEDLIFIAE   480
Harvard_(1A)       NAFRNVDGSGLVSKLIGLCKKIIPPTNIRENLYNRTASLTDLGGELCIKIKNEDLTFIAE   480
ATCC9441_(1B)      NAFRNVDGSGLVSKLIGLCKKIIPPTNIRENLYNRTASLTDLGGELCIKIKNEDLTFIAE   480
                   ******************************************************* ****

778-17_(2)         KNSFSEEPFQDETVSYNTKNKPLNFNYSLDKIILDYNLQSKITLPNDMTTPVTKGVLYIP   540
2017-061_(1H)      KNSFSEEPFQDETVSYNTKNKPLNFNYSLDKIILDYNLQSKITLPNDRTTPVTKGVLYIP   540
202_15_(1F)        KNSFSEEPFQDETVSYNTKNKPLNFNYSLDKIILDYNLQSKITLPNDRTTPVTKGVLYIP   540
512-15_(1F)        KNSFSEEPFQDETVSYNTKNKPLNFNYSLDKIILDYNLQSKITLPNDRTTPVTKGVLYIP   540
63-05_(2)          KNSFSEEPFQDETVSYNTKNKPLNFNYSLDKIILDYNLQSKITLPNDRTTPVTKGVLYIP   540
TMB2_(1B)          KNSFSEEPFQDETVSYNTKNKPLNFNYSIDKIILDYNLQSKITLPNDRTTPVTKGILYIP   540
3582_(1D)          KNSFSEEPFQDETVSYNTKNKPLNFNYSLDKIILDYNLQSKITLPNDRTTPVTKGVLYIP   540
1337_(1C)          KNSFSEEPFQDETVSYNTKNKPLNFNYSLDKIILDYNLQSKITLPNDRTTPVTKGVLYIP   540
```

```
12124569_(2)        KNSFSEEPFQDETVSYNTKNKPLNFNYSLDKIILDYNLQSKITLPNDRTTPVTKGIPYAP      540
132CV_(1G)          KNSFSEEPFQDEIVSYNTKNKPLNFNYSLDKIILDYNLQSKITLPNDRTTPVTKGIPYAP      540
Harvard_(1A)        KNSFSEEPFQDEIVSYNTKNKPLNFNYSLDKIIVDYNLQSKITLPNDRTTPVTKGIPYAP      540
ATCC9441_(1B)       KNSFSEEPFQDEIVSYNTKNKPLNFNYSLDKIILDYNLQSKITLPNDRTTPVTKGIPYAP      540
                    ************ ***************:****:************* *******: * *

778-17_(2)          KYKSNAASTIEIHNIDDNTIYQYLYAQKSPTTLQRITMTNSVDDALINSAKIYSYFPSLI      600
2017-061_(1H)       KYKSNAASTIEIHNIDDNTIYQYLYAQKSPTTLQRITMTNSVDDALINSAKIYSYFPSLI      600
202_15_(1F)         KYKSNAASTIEIHNIDDNTIYQYLYAQKSPTTLQRITMTNSVDDALINSAKIYSYFPSLI      600
512-15_(1F)         KYKSNAASTIEIHNIDDNTIYQYLYAQKSPTTLQRITMTNSVDDALINSAQIYSYFPSLI      600
63-05_(2)           KYKSNAASTIEIHNIDDNTIYQYLYAQKSPTTLQRITMTNSVDDALINSAKIYSYFPSVI      600
TMB2_(1B)           KYKSNAASTIEIHNIDDNTIYQYLYAQKSPTTLQKITMTNSVDDALINSTKIYSYFPSVI      600
3582_(1D)           KYKSNAASTIEIHNIDDNTIYQYLYAQKSPTTLQRITMTNSVDDALINSAKIYSYFPSVI      600
1337_(1C)           KYKSNAASTIEIHNIDDNTIYQYLYAQKSPTTLQRITMTNSVDDALINSAKIYSYFPSVI      600
12124569_(2)        KYKSNAASTIEIHNIDDNTIYQYLYAQKSPTTLQRITMTNSVDDALINSTKIYSYFPSVI      600
132CV_(1G)          EYKSNAASTIEIHNIDDNTIYQYLYAQKSPTTLQRITMTNSVDDALINSTKIYSYFPSVI      600
Harvard_(1A)        EYKSNAASTIEIHNIDDNTIYQYLYAQKSPTTLQRITMTNSVDDALINSTKIYSYFPSVI      600
ATCC9441_(1B)       EYKSNAASTIEIHNIDDNTIYQYLYAQKSPTTLQRITMTNSVDDALINSTKIYSYFPSVI      600
                    :*********************************:**************::*******:*

778-17_(2)          SKVNQGAQGILFLQWVRDIIDDFTNESSQKTTIDKISDVSIIVPYIGPALNIVKQGYEGN      660
2017-061_(1H)       SKVNQGAQGILFLQWVRDIIDDFTNESSQKTTIDKISDVSIIVPYIGPALNIVKQGYEGN      660
202_15_(1F)         SKVNQGAQGILFLQWVRDIIDDFTNESSQKTTIDKISDVSIIVPYIGPALNIVKQGYEGN      660
512-15_(1F)         SKVNQGAQGILFLQWVRDIIDDFTNESSQKTTIDKISDVSIIVPYIGPALNIVKQGYEGN      660
63-05_(2)           SKVNQGAQGILFLQWVRDIIDDFTNESSQKTTIDKISDVSIIVPYIGPALNIVKQGYEGN      660
TMB2_(1B)           SKVNQGAQGILFLQWVRDIIDDFTNESSQKTTIDKISDVSIIVPYIGPALNIVKQGYEGN      660
3582_(1D)           SKVNQGAQGILFLQWVRDIIDDFTNESSQKTTIDKISDVSIIVPYIGPALNIVKQGYEGN      660
1337_(1C)           SKVNQGAQGILFLQWVRDIIDDFTNESSQKTTIDKISDVSIIVPYIGPALNIVKQGYEGN      660
12124569_(2)        SKVNQGAQGILFLQWVRDIIDDFTNESSQKTTIDKISDVSTIVPYIGPALNIVKQGYEGN      660
132CV_(1G)          SKVNQGAQGILFLQWVRDIIDDFTNESSQKTTIDKISDVSTIVPYIGPALNIVKQGYEGN      660
Harvard_(1A)        SKVNQGAQGILFLQWVRDIIDDFTNESSQKTTIDKISDVSTIVPYIGPALNIVKQGYEGN      660
ATCC9441_(1B)       SKVNQGAQGILFLQWVRDIIDDFTNESSQKTTIDKISDVSTIVPYIGPALNIVKQGYEGN      660
                    **************************************** *******************

778-17_(2)          FIGALETTGVVLLLEYIPEITLPVIAALSIAESSTQKEKIIKTIDNFLEKRYEKWIEVYK      720
2017-061_(1H)       FIGALETTGVVLLLEYIPEITLPVIAALSIAESSTQKEKIIKTIDNFLEKRYEKWIEVYK      720
202_15_(1F)         FIGALETTGVVLLLEYIPEITLPVIAALSIAESSTQKEKIIKTIDNFLEKRYEKWIEVYK      720
512-15_(1F)         FIGALETTGVVLLLEYIPEITLPVIAALSIAESSTQKEKIIKTIDNFLEKRYEKWIEVYK      720
63-05_(2)           FIGALETTGVVLLLEYIPEITLPVIAALSIAESSTQKEKIIKTIDNFLEKRYEKWIEVYK      720
TMB2_(1B)           FIGALETTGVVLLLEYIPEITLPVIAALSIAESSTQKEKIIKTIDNFLEKRYEKWIEVYK      720
3582_(1D)           FIGALETTGVVLLLEYIPEITLPVIAALSIAESSTQKEKIIKTIDNFLEKRYEKWIEVYK      720
1337_(1C)           FIGALETTGVVLLLEYIPEITLPVIAALSIAESSTQKEKIIKTIDNFLEKRYEKWIEVYK      720
12124569_(2)        FIGALETTGVVLLLEYIPEITLPVIAALSIAESSTQKEKIIKTIDNFLEKRYEKWIEVYK      720
132CV_(1G)          FIGALETTGVVLLLEYIPEITLPVIAALSIAESSTQKEKIIKTIDNFLEKRYEKWIEVYK      720
Harvard_(1A)        FIGALETTGVVLLLEYIPEITLPVIAALSIAESSTQKEKIIKTIDNFLEKRYEKWIEVYK      720
ATCC9441_(1B)       FIGALETTGVVLLLEYIPEITLPVIAALSIAESSTQKEKIIKTIDNFLEKRYEKWIEVYK      720
                    ************************************************************

778-17_(2)          LVKAKWLGTVNTQFQKRSYQMYRSLEYQVDAIKKIIDYEYKIYSGPDKEQIADEINNLKN      780
2017-061_(1H)       LVKAKWLGTVNTQFQKRSYQMYRSLEYQVDAIKKIIDYEYKIYSGPDKEQIADEINNLKN      780
202_15_(1F)         LVKAKWLGTVNTQFQKRSYQMYRSLEYQVDAIKKIIDYEYKIYSGPDKEQIADEINNLKN      780
512-15_(1F)         LVKAKWLGTVNTQFQKRSYQMYRSLEYQVDAIKKIIDYEYKIYSGPDKEQIADEINNLKN      780
63-05_(2)           LVKAKWLGTVNTQFQKRSYQMYRSLEYQVDAIKKIIDYEYKIYSGPDKEQIADEINNLKN      780
TMB2_(1B)           LVKAKWLGTVNTQFQKRSYQMYRSLEYQVDAIKKIIDYEYKIYSGPDKEQIADEINNLKN      780
3582_(1D)           LVKAKWLGTVNTQFQKRSYQMYRSLEYQVDAIKKIIDYEYKIYSGPDKEQIADEINNLKN      780
1337_(1C)           LVKAKWLGTVNTQFQKRSYQMYRSLEYQVDAIKKIIDYEYKIYSGHDREQIADEINNLKN      780
12124569_(2)        LIKAKWLGTVNTQFQKRSYQMYRSLEYQVDAIKKIIDYEYKIYSGPDKEQIADEINNLKN      780
132CV_(1G)          LVKAKWLGTVNTQFQKRSYQMYRSLEYQVDAIKKIIDYEYKIYSGPDKEQIADEINNLKN      780
Harvard_(1A)        LVKAKWLGTVNTQFQKRSYQMYRSLEYQVDAIKKIIDYEYKIYSGPDKEQIADEINNLKN      780
ATCC9441_(1B)       LVKAKWLGTVNTQFQKRSYQMYRSLEYQVDAIKKIIDYEYKIYSGPDKEQIADEINNLKN      780
```

```
                    *:******************************************* *:************

778-17_(2)          KLEEKANKAMININIFMRESSRSFLVNQMINEAKKQLLEFDTQSKNILMQYIKANSKFIG      840
2017-061_(1H)       KLEEKANEAMININIFMKESSRSFLVNQMINQAKKQLLEFDTQSKNILMQYIKANSKFIG      840
202_15_(1F)         KLEEKANEAMININIFMKESSRSFLVNQMINQAKKQLLEFDTQSKNILMQYIKANSKFIG      840
512-15_(1F)         KLEEKANEAMININIFMKESSRSFLVNQMINQAKKQLLEFDTQSKNILMQYIKANSKFIG      840
63-05_(2)           KLEEKANKAMININIFMKESSRSFLVNQMINQAKKQLLEFDTQSKNILMQYIKANSKFIG      840
TMB2_(1B)           KLEEKANKAMININIFMRESSRSFLVNQMINEAKKQLLEFDTQSKNILMQYIKANSKFIG      840
3582_(1D)           KLEEKANKAMININIFMRESSRSFLVNQMINEAKKQLLEFDTQSKNILMQYIKANSKFIG      840
1337_(1C)           KLEEKANEAMININIFMRESSRSFLVNQMINQAKKQLLEFDTQSKNILMQYIKANSKFIG      840
12124569_(2)        KLEEKANKAMININIFMRESSRSFLVNQMINEAKKQLLEFDTQSKNILMQYIKANSKFIG      840
132CV_(1G)          KLEEKANKAMININIFMRESSRSFLVNQMINEAKKQLLEFDTQSKNILMQYIKANSKFIG      840
Harvard_(1A)        KLEEKANKAMININIFMRESSRSFLVNQMINEAKKQLLEFDTQSKNILMQYIKANSKFIG      840
ATCC9441_(1B)       KLEEKANKAMININIFMRESSRSFLVNQMINEAKKQLLEFDTQSKNILMQYIKANSKFIG      840
                    *******:*********:*************:****************************

778-17_(2)          ITELKKLESKINKVFSTPIPFSYSKNLDCWVDNEEDIDVILKKSTILNLDINNDIISDIS      900
2017-061_(1H)       ITELKKLESKINKVFSTPIPFSYSKNLDCWVDNEEDIDVILKKSTILNLDINNDIISDIS      900
202_15_(1F)         ITELKKLESKINKVFSTPIPFSYSKNLDCWVDNEEDIDVILKKSTILNLDINNDIISDIS      900
512-15_(1F)         ITELKKLESKINKVFSTPIPFSYSKNLDCWVDNEEDIDVILKKSTILNLDINNDIISDIS      900
63-05_(2)           ITELKKLESKINKVFSTPIPFSYSKNLDCWVDNEEDIDVILKKSTILNLDINNDIISDIS      900
TMB2_(1B)           ITELKKLESKINKVFSTPIPFSYSKNLDCWVDNEEDIDVILKKSTILNLDINNDIISDIS      900
3582_(1D)           ITELKKLESKINKVFSTPIPFSYSKNLDCWVDNEEDIDVILKKSTILNLDINNDIISDIS      900
1337_(1C)           ITELKKLESKINKVFSTPIPFSYSKNLDCWVDNEEDIDVILKKSTILNLDINNDIISDIS      900
12124569_(2)        ITELKKLESKINKVFSTPIPFSYSKNLDCWVDNEEDIDVILKKSTILNLDINNDIISDIS      900
132CV_(1G)          ITELKKLESKINKVFSTPIPFSYSKNLDCWVDNEEDIDVILKKSTILNLDINNDIISDIS      900
Harvard_(1A)        ITELKKLESKINKVFSTPIPFSYSKNLDCWVDNEEDIDVILKKSTILNLDINNDIISDIS      900
ATCC9441_(1B)       ITELKKLESKINKVFSTPIPFSYSKNLDCWVDNEEDIDVILKKSTILNLDINNDIISDIS      900
                    ************************************************************

778-17_(2)          GFNSSVITYPDAQLVPGINGKAIHLVNNESSEVIVHKAMDIEYNDMFNNFTVSFWLRVPK      960
2017-061_(1H)       GFNSSVITYPDAQLVPGINGKAIHLVNNESSEVIVHKAMDIEYNDMFNNFTVSFWLRVPK      960
202_15_(1F)         GFNSSVITYPDAQLVPGINGKAIHLVNNESSEVIVHKAMDIEYNDMFNNFTVSFWLRVPK      960
512-15_(1F)         GFNSSVITYPDAQLVPGINGKAIHLVNNESSEVIVHKAMDIEYNDMFNNFTVSFWLRVPK      960
63-05_(2)           GFNSSVITYPDAQLVPGINGKAIHLVNNESSEVIVHKAMDIEYNDMFNNFTVSFWLRVPK      960
TMB2_(1B)           GFNSSVITYPDAQLVPGINGKAIHLVNNESSEVIVHKAMDIEYNDMFNNFTVSFWLRVPK      960
3582_(1D)           GFNSSVITYPDAQLVPGINGKAIHLVNNESSEVIVHKAMDIEYNDMFNNFTVSFWLRVPK      960
1337_(1C)           GFNSSVITYPDAQLVPGINGKAIHLVNNESSEVIVHKAMDIEYNDMFNNFTVSFWLRVPK      960
12124569_(2)        GFNSSVITYPDAQLVPGINGKAIHLVNNESSEVIVHKAMDIEYNDMFNNFTVSFWLRVPK      960
132CV_(1G)          GFNSSVITYPDAQLVPGINGKAIHLVNNESSEVIVHKAMDIEYNDMFNNFTVSFWLRVPK      960
Harvard_(1A)        GFNSSVITYPDAQLVPGINGKAIHLVNNESSEVIVHKAMDIEYNDMFNNFTVSFWLRVPK      960
ATCC9441_(1B)       GFNSSVITYPDAQLVPGINGKAIHLVNNESSEVIVHKAMDIEYNDMFNNFTVSFWLRVPK      960
                    ************************************************************

778-17_(2)          VSASHLEQYGTNEYSIISSMKKYSLSIGSGWSVSLKGNNLIWTLKDSAGEVRQITFSDLR      1020
2017-061_(1H)       VSASHLEQYGTNEYSIISSMKKYSLSIGSGWSVSLKGNNLIWTLKDSAGEVRQITFRDLP      1020
202_15_(1F)         VSASHLEQYGTNEYSIISSMKKYSLSIGSGWSVSLKGNNLIWTLKDSAGEVRQITFSDLR      1020
512-15_(1F)         VSASHLEQYGTNEYSIISSMKKYSLSIGSGWSVSLKGNNLIWTLKDSAGEVRQITFSDLR      1020
63-05_(2)           VSASHLEQYGTNEYSIISSMKKYSLSIGSGWSVSLKGNNLIWTLKDSAGEVRQITFRDLS      1020
TMB2_(1B)           VSASHLEQYGTNEYSIISSMKKYSLSIGSGWSVSLKGNNLIWTLKDSAGEVRQITFRDLS      1020
3582_(1D)           VSASHLEQYGTNEYSIISSMKKYSLSIGSGWSVSLKGNNLIWTLKDSAGEVRQITFRDLS      1020
1337_(1C)           VSASHLEQYGTNEYSIISSMKKYSLSIGSGWSVSLKGNNLIWTLKDSAGEVRQITFRDLS      1020
12124569_(2)        VSASHLEQYGTNEYSIISSMKKYSLSIGSGWSVSLKGNNLIWTLKDSAGEVRQITFSDLS      1020
132CV_(1G)          VSASHLEQYDTNEYSIISSMKKYSLSIGSGWSVSLKGNNLIWTLKDSAGEVRQITFRDLS      1020
Harvard_(1A)        VSASHLEQYGTNEYSIISSMKKHSLSIGSGWSVSLKGNNLIWTLKDSAGEVRQITFRDLP      1020
ATCC9441_(1B)       VSASHLEQYGTNEYSIISSMKKYSLSIGSGWSVSLKGNNLIWTLKDSAGEVRQITFRDLS      1020
                    *********.************:********************************* **

778-17_(2)          DKFNAYLANKWVFITITNDRLSSANLYINGVLMKNAEITGLGAIREDNNITLKLDRCNNN      1080
2017-061_(1H)       DKFNAYLANKWVFITITNDRLSSANLYINGVLMGSAEITGLGAIREDNNITLKLDRCNNN      1080
```

```
202_15_(1F)         DKFNAYLANKWVFITITNDRLSSTNLYINGVLMKNAEITGLGAIREDNNITLKLDRCNNN      1080
512-15_(1F)         DKFNAYLANKWVFITITNDRLSSTNLYINGVLMKNAEITGLGAIREDNNITLKLDRCNNN      1080
63-05_(2)           DKFNAYLANKWVFITITNDRLSSANLYINGVLMESAEITGLGAIREDNNITLKLDRCNNN      1080
TMB2_(1B)           DKFNAYLANKWVFITITNDRLSSANLYINGVLMGSAEITGLGAIREDNNITLKLDRCNNN      1080
3582_(1D)           DKFNAYLANKWVFITITNDRLSSANLYINGVLMGSAEITGLGAIREDNNITLKLDRCNNN      1080
1337_(1C)           DKFNAYLANKWVFITITNDRLSSANLYINGVLMGSAEITGLGAIREDNNITLKLDRCNNN      1080
12124569_(2)        DKFNAYLANKWVFITITNDRLSSANLYINGVLMKNAEITGLGAIREDNNITLKLDRCNNN      1080
132CV_(1G)          DKFNAYLANKWVFITITNDRLSSANLYINGVLMGSAEITGLGAIREDNNITLKLDRCNNN      1080
Harvard_(1A)        DKFNAYLANKWVFITITNDRLSSANLYINGVLMGSAEITGLGAIREDNNITLKLDRCNNN      1080
ATCC9441_(1B)       DKFNAYLANKWVFITITNDRLSSANLYINGVLMGSAEITGLGAIREDNNITLKLDRCNNN      1080
                    ***********************:********* .*************************

778-17_(2)          NQYVSIDKFRIFCKALNPKEIEKLYTSYLSITFLRDFWGNPLRYDTEYYLIPVDSSSNSE      1140
2017-061_(1H)       NQYVSIDKFRIFCKALNPKEIEKLYTSYLSITFLRDFWGNPLRYDTEYYLIPVDSSSNSE      1140
202_15_(1F)         NQYVSIDKFRIFCKALNPKEIEKLYTSYLSITFLRDFWGNPLRYDTEYYLIPVDSSSNSE      1140
512-15_(1F)         NQYVSIDKFRIFCKALNPKEIEKLYTSYLSITFLRDFWGNPLRYDTEYYLIPVDSSSNSE      1140
63-05_(2)           NQYVSIDKFRIFCKALNPKEIEKLYTSYLSITFLRDFWGNPLRYDTEYYLIPVASSS---      1137
TMB2_(1B)           NQYVSIDKFRIFCKALNPKEIEKLYTSYLSITFLRDFWGNPLRYDTEYYLIPVASSS---      1137
3582_(1D)           NQYVSIDKFRIFCKALNPKEIEKLYTSYLSITFLRDFWGNPLRYDTEYYLIPVASSS---      1137
1337_(1C)           NQYVSIDKFRIFCKALNPKEIEKLYTSYLSITFLRDFWGNPLRYDTEYYLIPVASSS---      1137
12124569_(2)        NQYVSIDKFRIFCKALNPKEIEKLYTSYLSITFLRDFWGNPLRYDTEYYLIPVASSS---      1137
132CV_(1G)          NQYVSIDKFRIFCKALNPKEIEKLYTSYLSITFLRDFWGNPLRYDTEYYLIPVAYSS---      1137
Harvard_(1A)        NQYVSIDKFRIFCKALNPKEIEKLYTSYLSITFLRDFWGNPLRYDTEYYLIPVASSS---      1137
ATCC9441_(1B)       NQYVSIDKFRIFCKALNPKEIEKLYTSYLSITFLRDFWGNPLRYDTEYYLIPVASSS---      1137
                    *****************************************************  **

778-17_(2)          YKDIQLKNITDYMYLTNAPSYTNGKLNIYYRRLYNGLKFIIKRYTPNNEIDSFVKSGDFI      1200
2017-061_(1H)       SKDIQLKNITDYMYLTTAPSYTNGKLNIYYRRLYNGLKFIIKRYTPNNEIDSFVRSGDFI      1200
202_15_(1F)         SKDIQLKNITDYMYLTNAPSYTNGKLNIYYRRLYNGLKFIIKRYTPNNEIDSFVKSGDFI      1200
512-15_(1F)         SKDIQLKNITDYMYLTNAPSYTNGKLNIYYRRLYNGLKFIIKRYTPNNEIDSFVKSGDFI      1200
63-05_(2)           -KDVQLKNITDYMYLTNAPSYTNGKLNIYYRRLYNGLKFIIKRYTPNNEIDSFVRSGDFI      1196
TMB2_(1B)           -KDVQLKNITDYMYLTNAPSYTNGKLNIYYRRLYNGLKFIIKRYTPNNEIDSFVRSGDFI      1196
3582_(1D)           -KDVQLKNITDYMYLTNAPSYTNGKLNIYYRRLYNGLKFIIKRYTPNNEIDSFVKSGDFI      1196
1337_(1C)           -KDVQLKNITDYMYLTNAPSYTNGKLNIYYRRLYSGLKFIIKRYTPNNEIDSFVKSGDFI      1196
12124569_(2)        -KDVQLKNITDYMYLTNAPSYTNGKLNIYYRRLYSGLKFIIKRYTPNNEIDSFVKSGDFI      1196
132CV_(1G)          -KDVQLKNITDYMYLTNAPSYTNGKLNIYYRRLYSGLKFIIKRYTPNNEIDSFVRSGDFI      1196
Harvard_(1A)        -KDVQLKNITDYMYLTNAPSYTNGKLNIYYRRLYNGLKFIIKRYTPNNEIDSFVKSGDFI      1196
ATCC9441_(1B)       -KDVQLKNITDYMYLTNAPSYTNGKLNIYYRRLYNGLKFIIKRYTPNNEIDSFVKSGDFI      1196
                     **:************.*****************.*******************:*****

778-17_(2)          KLYVSYNNNEHIVGYPKDGNAFNNSDRILRVGYNAPGIPLYKKMEAVKLRDLKTYSVQLK      1260
2017-061_(1H)       KLYVSYNNNEHIVGYPKDGNAFNNLDRILRVGYNAPGIPLYKKMEAVKLRDLKTYSVQLK      1260
202_15_(1F)         KLYVSYNNNEHIVGYPKDGNAFNNSDRILRVGYNAPGIPLYKKMEAVKLRDLKTYSVQLK      1260
512-15_(1F)         KLYVSYNNNEHIVGYPKDGNAFNNSDRILRVGYNAPGIPLYKKMEAVKLRDLKTYSVQLK      1260
63-05_(2)           KLYVSYNNNEHIVGYPKDGNAFNNLDRILRVGYNAPGIPLYKKMEAVKLRDLKTYSVQLK      1256
TMB2_(1B)           KLYVSYNNNEHIVGYPKDGNAFNNLDRILRVGYNAPGIPLYKKMEAVKLRDLKTYSVQLK      1256
3582_(1D)           KLYVSYNNNEHIVGYPKDGNAFNNLDRILRVGYNAPGIPLYKKMEAVKLRDLKTYSVQLK      1256
1337_(1C)           KLYVSYNNNEHIVGYPKDGNAFNNLDRILRVGYNAPGIPLYKKMEAVKLRDLKTYSVQLK      1256
12124569_(2)        KLYVSYNNNEHIVGYPKDGNAFNNLDRILRVGYNAPGIPLYKKMEAVKLRDLKTYSVQLK      1256
132CV_(1G)          KLYVSYNNNEHIVGYPKDGNAFNNLDRILRVGYNAPGIPLYKKMEAVKLRDLKTYSVQLK      1256
Harvard_(1A)        KLYVSYNNNEHIVGYPKDGNAFNNLDRILRVGYNAPGIPLYKKMEAVKLRDLKTYSVQLK      1256
ATCC9441_(1B)       KLYVSYNNNEHIVGYPKDGNAFNNLDRILRVGYNAPGIPLYKKMEAVKLRDLKTYSVQLK      1256
                    ************************ ***********************************

778-17_(2)          LYDDKDASLGLVGTHNGQIGNDPDRDILIASNWYFNHLKDKTLTCDWYFVPTDEGWTND-      1319
2017-061_(1H)       LYDDKDASLGLVGTHNGQIGNDPNRDILIASNWYFNHLKDKTLTCDWYFVPTDEGWTND-      1319
202_15_(1F)         LYDDKNASLGLVGIHNGQIGNDPKRDILIASNWYFNHLKDKTLTCDWYFVPTDEGWTND-      1319
512-15_(1F)         LYDDKNASLGLVGIHNGQIGNDPKRDILIASNWYFNHLKDKTLTCDWYFVPTDEGWTND-      1319
63-05_(2)           LYDDKNASLGLVGIRNGQIGNDPNRDILIASNWYFNHLKDKTLTCDWYFVPTDEGWTND-      1315
TMB2_(1B)           LYDDKNASLGLVGTHNGQIGNDPNRDILIASNWYFNHLKNKTLGCDWYFVPTDEGWTNDK      1316
```

```
3582_(1D)           LYDDKNASLGLVGTHNGQIGNDPNRDILIASNWYFNHLKDKILGCDWYFVPTDEGWTND-      1315
1337_(1C)           LYDDKNASLGLVGTHNGQIGNDPNRDILIASNWYFNHLKDKILGCDWYFVPTDEGWTND-      1315
12124569_(2)        LYDDKNASLGLVGIRNGQIGNDPNRDILIASNWYFNHLKDKTLTCDWYFVPTDEGWTND-      1315
132CV_(1G)          LYDDKDASLGLVGTHNGQIGNDPNRDILIASNWYFNHLKDKTLTCDWYFVPTDEGWTND-      1315
Harvard_(1A)        LYDDKNASLGLVGTHNGQIGNDPNRDILIASNWYFNHLKDKILGCDWYFVPTDEGWTND-      1315
ATCC9441_(1B)       LYDDKNASLGLVGTHNGQIGNDPNRDILIASNWYFNHLKDKILGCDWYFVPTDEGWTND-      1315
                    *****:******* :********.***************:* * ***************

778-17_(2)          --    1319
2017-061_(1H)       --    1319
202_15_(1F)         --    1319
512-15_(1F)         --    1319
63-05_(2)           --    1315
TMB2_(1B)           ID    1318
3582_(1D)           --    1315
1337_(1C)           --    1315
12124569_(2)        --    1315
132CV_(1G)          --    1315
Harvard_(1A)        --    1315
ATCC9441_(1B)       --    1315
```

Figure S2: Alignment of TeNT protein sequences showing an insertion of four amino acids obtained by PCR amplification of *tent* and sequencing. The 3' part of *tent* was PCR amplified with P2302 (CTGCAGTTAATCATTTGTCCATCCTTC) and P2394 (GATATTATATCAGATATATCTGGG), cloned in pCR2 and sequenced by the Sanger method. The deduced amino acid sequences are shown. The amino acid insertion in *C. tetani* strains 2017.61, 202.15, and 358.99 is highlighted in yellow.

```
CN655      -DIISDISGFNSSVITYPDAQLVPGINGKAIHLVNNESSEVIVHKAMDIEYNDMFNNFTVSFWLRVPKVSASHLEQYGTN
2017.061   -DIISDISGFNSSVITYPDAQLVPGINGKAIHLVNNESSEVIVHKAMDIEYNDMFNNFTVSFWLRVPKVSASHLEQYGTN
202.15     -DIISDISGFNSSVITYPDAQLVPGINGKAIHLVNNESSEVIVHKAMDIEYNDMFNNFTVSFWLRVPKVSASHLEQYGTN
358.99     -DIISDISGFNSSVITYPDAQLVPGINGKAIHLVNNESSEVIVHKAMDIEYNDMFNNFTVSFWLRVPKVSASHLEQYGTN

CN655       EYSIISSMKKHSLSIGSGWSVSLKGNNLIWTLKDSAGEVRQITFRDLPDKFNAYLANKWVFITITNDRLSSANLYINGVL
2017.061    EYSIISSMKKYSLSIGSGWSVSLKGNNLIWTLKDSAGEVRQITFRDLPDKFNAYLANKWVFITITNDRLSSANLYINGVL
202.15      EYSIISSMKKYSLSIGSGWSVSLKGNNLIWTLKDSAGEVRQITFSDLRDKFNAYLANKWVFITITNDRLSSTNLYINGVL
358.99      EYSIISSMKKYSLSIGSGWSVSLKGNNLIWTLKDSAGEVRQITFSDLRDKFNAYLANKWVFITITNDRLSSTNLYINGVL

CN655       MGSAEITGLGAIREDNNITLKLDRCNNNNQYVSIDKFRIFCKALNPKEIEKLYTSYLSITFLRDFWGNPLRYDTEYYLIP
2017.061    MGSAEITGLGAIREDNNITLKLDRCNNNNQYVSIDKFRIFCKALNPKEIEKLYTSYLSITFLRDFWGNPLRYDTEYYLIP
202.15      MKNAEITGLGAIREDNNITLKLDRCNNNNQYVSIDKFRIFCKALNPKEIEKLYTSYLSITFLRDFWGNPLRYDTEYYLIP
358.99      MKNAEITGLGAIREDNNITLKLDRCNNNNQYVSIDKFRIFCKALNPKEIEKLYTSYLSITFLRDFWGNPLRYDTEYYLIP

CN655       VASS----SKDVQLKNITDYMYLTNAPSYTNGKLNIYYRRLYNGLKFIIKRYTPNNEIDSFVKSGDFIKLYVSYNNNEHI
2017.061    VDSSSNSESKDIQLKNITDYMYLTTAPSYTNGKLNIYYRRLYNGLKFIIKRYTPNNEIDSFVRSGDFIKLYVSYNNNEHI
202.15      VDSSSNSESKDIQLKNITDYMYLTNAPSYTNGKLNIYYRRLYNGLKFIIKRYTPNNEIDSFVKSGDFIKLYVSYNNNEHI
358.99      VDSSSNSESKDIQLKNITDYMYLTNAPSYTNGKLNIYYRRLYNGLKFIIKRYTPNNEIDSFVKSGDFIKLYVSYNNNEHI

CN655       VGYPKDGNAFNNLDRILRVGYNAPGIPLYKKMEAVKLRDLKTYSVQLKLYDDKNASLGLVGTHNGQIGNDPNRDILIASN
2017.061    VGYPKDGNAFNNLDRILRVGYNAPGIPLYKKMEAVKLRDLKTYSVQLKLYDDKDASLGLVGTHNGQIGNDPNRDILIASN
202.15      VGYPKDGNAFNNSDRILRVGYNAPGIPLYKKMEAVKLRDLKTYSVQLKLYDDKNASLGLVGIHNGQIGNDPKRDILIASN
358.99      VGYPKDGNAFNNSDRILRVGYNAPGIPLYKKMEAVKLRDLKTYSVQLKLYDDKNASLGLVGIHNGQIGNDPKRDILIASN

CN655       WYFNHLKDKILGCDWYFVPTDEGWTND*  1315
2017.061    WYFNHLKDKTLTCDWYFVPTDEGWTND*  1319
202.15      WYFNHLKDKTLTCDWYFVPTDEGWTND*  1319
358.99      WYFNHLKDKTLTCDWYFVPTDEGWTND*  1319
```
